# Exosomes delivering a high-throughput-screened RNA polymerase inhibitor for highly effective therapy of multidrug-resistant bacterial infected pneumonia

**DOI:** 10.1016/j.bioactmat.2026.07.023

**Published:** 2026-07-16

**Authors:** Yiming Xiang, Ziya Gong, Juying Liu, Yizhou Zhu, Congyang Mao, Can Ai, Chaofeng Wang, Xiaofei Yang, Xiangmei Liu, Kelvin W.K. Yeung, Shuilin Wu

**Affiliations:** a Hubei Key Laboratory of Medical Information Analysis and Tumor Diagnosis & Treatment, Key Laboratory of Cognitive Science, College of Biomedical Engineering, South-Central Minzu University, Wuhan, 430074, China; b Department of Orthopaedics & Traumatology, Li Ka Shing Faculty of Medicine, The University of Hong Kong, Pokfulam, Hong Kong, 999077, China; c School of Life Science and Health Engineering, Hebei University of Technology, Tianjin, 300401, China; d School of Materials Science and Engineering, Peking University, Beijing, 100871, China

**Keywords:** Antibacterial, High-throughput screening, Exosome-based nanoplatform, Bacterial pneumonia, RNA polymerase inhibitor

## Abstract

Bacterial pneumonia remains a primary global health threat, necessitating the development of novel therapeutic strategies to overcome escalating antibiotic resistance. In this study, we identified a highly active bacterial RNAP inhibitor, the repurposed semi-synthetic anthracycline hydrochloride Epirubicin (EPI), via the in silico high-throughput screening of a commercial library containing 16,563 small molecules. We identified EPI as a potent multi-target antibacterial agent. Alongside its intrinsic DNA intercalation properties, EPI heavily interferes with RNAP by targeting conserved catalytic residues (LYS838 and ASP1003), destabilizing the RNAP structure through a mechanism distinct from rifampicin. *In vitro*, EPI (8 μg/mL) achieved 99% eradication of *S. aureus* and multidrug-resistant (MDR) *E. coli* within 12 h by disrupting carbohydrate metabolism and ATP synthesis. To enhance clinical efficacy, EPI was encapsulated in stem cell-derived exosomes (Exo/EPI). In murine pneumonia models, the Exo/EPI nanoplatform cleared 99% of bacteria within 12 h. Furthermore, the platform rapidly attenuated infection-induced pulmonary inflammation, evidenced by a marked reduction in inflammatory cell infiltration, while significantly accelerating structural lung tissue repair and preventing cellular apoptosis via organized collagen deposition. This integrated strategy of computational discovery and exosomes delivery provides a blueprint for developing multifunctional antimicrobials against MDR infections.

## Highlights

• EPI identified via high-throughput screening as a novel bacterial RNAP inhibitor.
• EPI binds Lys838/Asp1003 to disrupt RNAP structure and transcription.
• Exo/EPI nanoplatform achieves 99% bacterial clearance in bacterial infected pneumonia.

## Graphical abstract

Schematic illustration of the engineered Exo/EPI nanoplatform for the synergistic "kill-repair" therapy of multidrug-resistant (MDR) pneumonia. The workflow encompasses the high-throughput screening (HTS) of 16,563 natural products against bacterial RNA polymerase (RNAP), the identification of the lead compound EPI, and its subsequent encapsulation into mesenchymal stem cell (MSC)-derived exosomes to facilitate targeted pulmonary delivery, bacterial clearance (99%), and immunomodulatory tissue regeneration.

**Figure:**
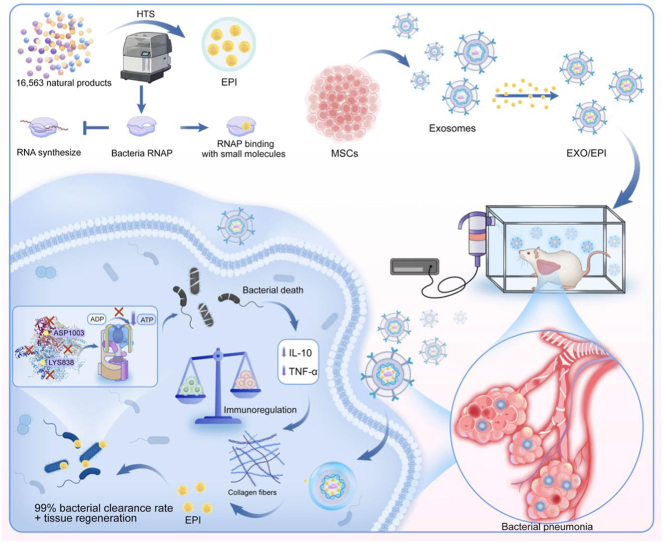

## 1 Introduction

Bacterial pneumonia remains a leading cause of infectious mortality worldwide, accounting for ∼80% of adult pneumonia cases despite widespread antibiotic use [[1], [2], [3], [4], [5]]. While *Streptococcus pneumoniae* and *Haemophilus influenzae* remain the most prevalent pathogens in community-acquired settings, the highest mortality and most severe structural lung damage are increasingly driven by multidrug-resistant (MDR) nosocomial pathogens [6,7]. Specifically, hospital-acquired pneumonia (HAP) and ventilator-associated pneumonia (VAP) are overwhelmingly dominated by *Staphylococcus aureus* (including MRSA) and MDR Gram-negative bacilli, such as *Escherichia coli* [8]. These highly refractory infections frequently precipitate severe tissue necrosis and inflammatory collapse, underscoring an urgent clinical mandate for advanced therapeutic platforms capable of not only eradicating resistant pathogens but also actively driving extracellular matrix repair and tissue regeneration. Due to escalating antibiotic resistance, bacterial pneumonia has maintained high morbidity and mortality rates since the 1960s [[9], [10], [11]]. Vulnerable populations (children, the elderly) increasingly suffer from refractory infections [[12], [13], [14]]. Antimicrobials remain the mainstay of treatment for bacterial pneumonia; however, the widespread overuse of antibiotics has driven the emergence of multidrug-resistant (MDR) pathogens, leading to a rising incidence of severe, intractable clinical infections [15,16]. The emergence of a large number of drug-resistant bacteria, including methicillin-resistant *Staphylococcus aureus* and multidrug-resistant *Escherichia coli*, has led to an urgent need for the development of novel antimicrobial drugs to reduce the emergence of drug resistance in order to efficiently treat bacterial pneumonia [17,18].

Computerized high-throughput drug screening accelerates drug discovery by enabling rapid molecular-level candidate identification [[19], [20], [21], [22], [23], [24]]. Recent advances leverage molecular docking against bacterial targets [25,26], significantly reducing development timelines while predicting pharmacokinetic properties [[27], [28], [29], [30]]. Bacterial RNA polymerase (RNAP) is an enzyme responsible for catalyzing the synthesis of three types of RNA (messenger RNA, ribosomal RNA and transporter RNA) in bacteria [[31], [32], [33]]. This enzyme is relatively large, and its core enzyme consists of several subunits with a structure usually α2ββ′ω, where the α2 subunit is responsible for the recognition of the regulatory factors, the β-subunit has the activity of a polymerase and is responsible for catalyzing RNA synthesis, and the β′ subunit is bound to the DNA binding [[34], [35], [36]]. The function of RNAP lies mainly in transcription, i.e. the transcription of template DNA into RNA using a DNA strand as a template using four ribonucleoside triphosphates as raw materials [37,38]. This process involves the RNA polymerase catalyzing the reaction by interacting with the DNA template sequence and using its active center to link the ribonucleoside triphosphates with the 3′-OH end of the RNA strand to form the RNA chain [[39], [40], [41]]. Bacterial RNAP is the core essential enzyme responsible for transcription, effectively catalyzing the synthesis of RNA from a DNA template [42,43]. Designing compounds or drugs by molecular docking to directly inhibit the activity of bacterial RNAP and block its transcription process to the DNA template, thus preventing bacterial gene expression and protein synthesis is a promising strategy for antibacterial drug development [44]. Furthermore, bacterial RNAP represents a highly advantageous, selectively toxic therapeutic target due to the profound structural divergence between prokaryotic and eukaryotic transcriptional machinery. Bacteria rely on a single, highly conserved RNAP enzyme featuring a relatively simple, five-subunit core architecture (α2ββ'ω) that requires transient σ factors for promoter recognition [32]. In stark contrast, mammalian cells utilize three distinct, highly complex polymerases (Pol I, II, and III), with the mRNA-synthesizing Pol II comprising up to 12 core subunits and requiring entirely different suites of general transcription factors [45]. Because the deep catalytic clefts and specific subunit interfaces of bacterial RNAP lack high structural homology with human RNA polymerases, small molecules designed to specifically bind bacterial RNAP can exert potent antimicrobial activity without directly poisoning the host's transcriptional apparatus.

**Fig. 1:**
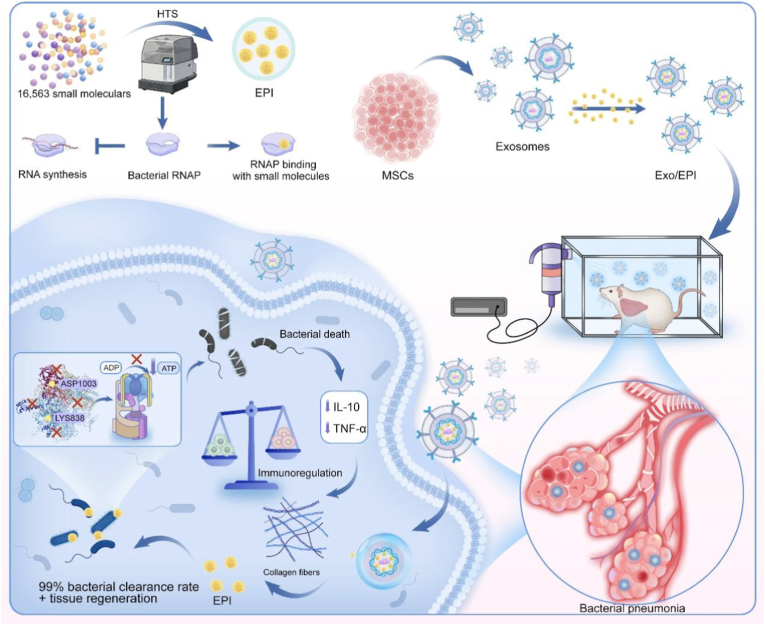
Schematic illustration of the Exo/EPI nanoplatform preparation and its dual-functional therapeutic mechanism in the lung microenvironment. The schematic simulates the extracellular alveolar space during severe acute bacterial pneumonia. Upon administration into this pulmonary microenvironment, the Exo/EPI platform acts extracellularly to rapidly eradicate multidrug-resistant bacteria, while simultaneously promoting structural tissue regeneration and collagen deposition in the surrounding damaged pulmonary stroma. The workflow encompasses the high-throughput screening (HTS) of 16,563 small molecules against bacterial RNA polymerase (RNAP), the identification of the lead compound EPI, and its subsequent encapsulation into mesenchymal stem cell (MSC)-derived exosomes to facilitate localized pulmonary delivery, bacterial clearance (99%), and immunomodulatory tissue regeneration.

Current limitations of RNAP-targeted drugs center on susceptibility to resistance mechanisms and lack of tissue-repair capabilities. While inhibiting RNAP disrupts bacterial transcription [[37], [38], [39], [40], [41]], clinical translation requires both novel compounds evading established resistance pathways and delivery systems mitigating infection-induced tissue damage. Herein, we propose an integrated strategy combining silico RNAP inhibitor discovery with exosome-mediated delivery. We hypothesize that combining RNAP inhibition with exosome-mediated delivery achieves synergistic kill-repair efficacy against drug-resistant pneumonia. Through high-throughput screening of 16,563 compounds against RNAP (PDB ID: 3DXJ), we identified hydrochloride Epirubicin (EPI), a novel inhibitor binding conserved residues (LYS838/ASP1003) distinct from rifampicin's site. EPI was further engineered into umbilical cord stem cell exosomes (Exo/EPI) to simultaneously eradicate pathogens and promote lung regeneration. This approach addresses the dual challenge of antibiotic resistance and tissue damage in pneumonia therapy (Fig. 1).

## 2 Results and discussion

### 2.1 Computer aided screening and *in vitro* antibacterial activity

**Fig. 2:**
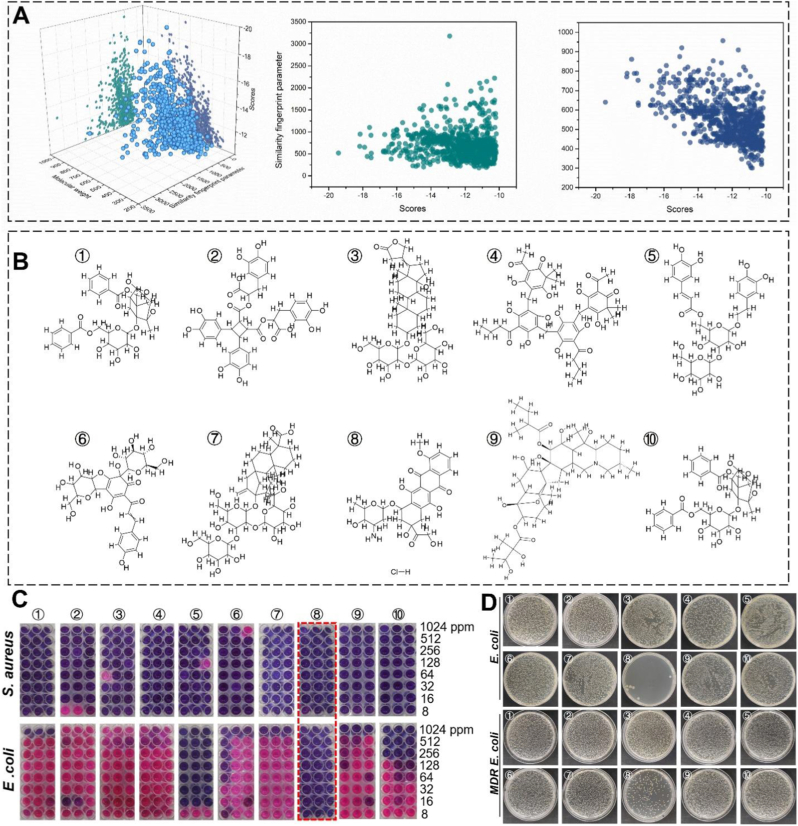
Identification and *in vitro* antimicrobial validation of lead RNAP inhibitors. (A) Three-dimensional scatter plots correlating molecular weight, similarity fingerprints, and docking scores for the screened library. (B) Chemical structures of the top 10 candidate compounds, highlighting Hydrochloride Epirubicin (EPI, Compound 8) as the primary lead. (C) Resazurin-based metabolic activity assays for *S. aureus* and *E. coli* treated with candidates at concentrations ranging from 8 to 1024 ppm. (D) Representative plate spread images demonstrating the superior bactericidal efficacy of EPI against standard and MDR *E. coli* strains.

Initial screening of 16,563 compounds yielded 767 candidates, which were analyzed using binary fingerprints to evaluate RNAP affinity. It can be found that most of the compounds have poor affinity with RNAP, only a few have theoretical inhibitory activity of RNAP, and the affinity for compounds whose molecular weights are too large or too small is low (Fig. 2A). This is mainly due to the fact that large molecular weight compounds are difficult to change the structure of the residues within the pocket after contacting with RNAP, whereas small molecular weight compounds have a weak intermolecular force with RNAP due to their small size and simple structure, which makes it difficult to form a stable complex structure and thus have a low inhibitory performance against RNAP. Further, 10 compounds with RNAP inhibitory activity were initially screened by affinity results, which were benzoylpaeoniflorin; salic acid; dryocrassin; glucuronidin; plantainoside; vicenin-2; stevioside; EPI); newcombatin; cannabinamide D (Fig. 2B). Through this virtual screening, 10 candidate compounds with high predicted RNAP binding affinities were initially selected. Recognizing that in silico binding affinity does not inherently translate to functional enzymatic inhibition, we subsequently subjected these 10 candidates to rigorous *in vitro* biological evaluations. To empirically determine their true antibacterial activity, standard plate spread assays and Minimum Inhibitory Concentration (MIC) determinations were performed against both standard and multidrug-resistant bacterial strains. Among the candidates, only EPI demonstrated profound, dose-dependent bactericidal efficacy, confirming that its computational affinity successfully translated into robust *in vitro* biological activity.

According to the results of resazurin staining, it can be found that these 10 compounds have good antibacterial effect on *S. aureus*, killing most of the bacteria at different concentrations (Fig. 2C). However, for the antimicrobial assay of *E. coli*, only EPI (Compound 8) and Compound 5 showed superior antibacterial effect, and EPI still had excellent antibacterial effect at the lowest concentration of 8 μg/mL. In particular, the antibacterial effect of EPI was more pronounced against *E. coli*. This suggests that among the 10 compounds, compound EPI had the best RNA polymerase inhibitory activity. We proceeded to verify this result by the results of plate spread. As shown in Fig. 2D, it shows the antibacterial results of the 10 compounds against *E. coli* and multidrug-resistant *E. coli* (MDR *E. coli*), respectively. To rigorously quantify this bactericidal activity, we performed standardized plate spread assays (CFU counting). The quantitative analysis demonstrated that EPI, at a minimal concentration of 8 μg/mL, achieved greater than a 3-log_10_ reduction (>99.9% bactericidal efficacy) against both standard *S. aureus* and MDR *E. coli* within 12 h of co-incubation (Fig. S1A). To quantitatively establish the baseline antimicrobial potency of the lead compound 8 (EPI), standard MIC assays were conducted. The MIC values for EPI were determined to be 2 μg/mL against *S. aureus*, 8 μg/mL against standard *E. coli*, and 16 μg/mL against the multidrug-resistant strain *MDR E. coli* (Fig. S1B). These robust inhibitory parameters provided the necessary pharmacological foundation for subsequent time-kill and *in vivo* evaluations. This confirms that EPI acts as a highly potent, rapid bactericidal agent, whereas the other screened compounds demonstrated insufficient quantitative reductions against these severe pathogens. The results of the plate coating showed that only compound EPI had a better antibacterial effect, however, several other screened compounds had insufficient antibacterial performance against *E. coli* and MDR *E. coli*.

While the data demonstrates differential antibacterial efficacy across species, the highly conserved nature of bacterial RNAP suggests the core molecular target remains identical. Therefore, this phenotypic variance is predominantly governed by the profound structural divergence in their respective cell envelopes, which dictates intracellular drug accumulation. The complex lipopolysaccharide (LPS) outer membrane and active efflux pump systems of Gram-negative *E. coli* present a fundamentally different permeability barrier compared to the thick, single-membrane peptidoglycan layer of Gram-positive *S. aureus.* Consequently, the specific physicochemical properties of the EPI molecule dictate its penetration kinetics through these distinct barriers, resulting in the observed variations in functional bactericidal concentrations.

### 2.2 Molecular docking elucidation and mechanistic evaluation of EPI-mediated bactericidal activity

**Fig. 3:**
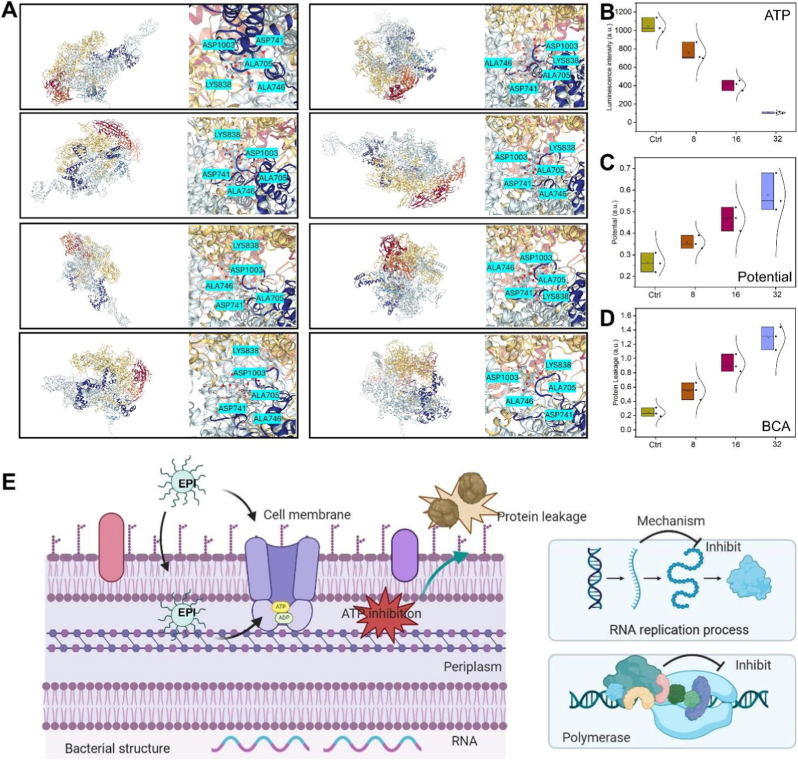
Computational and biochemical elucidation of the antimicrobial mechanism of EPI. (A) Representative molecular docking conformations illustrating the hydrogen bonding network between EPI and conserved RNAP residues, including Lys838 and Asp1003. (B-D) Quantitative analysis of bacterial physiological perturbations following EPI treatment: (B) concentration-dependent inhibition of ATP synthesis, (C) alterations in bacterial membrane potential, and (D) BCA assay-quantified efflux of intramembrane proteins indicating membrane compromise. (E) Proposed antimicrobial mechanism involving RNAP inhibition, metabolic exhaustion, and cytoplasmic leakage.

We had confirmed that compound EPI exhibits significant antibacterial properties even at extremely low concentrations. To further explore the potential antibacterial mechanism of EPI, we conducted a molecular docking study to analyze the interaction between compound EPI and RNAP, particularly focusing on the nature of their interaction and the possible inhibitory principle. It can be seen that upon contact between RNAP and EPI, eight possible conformations exist for the complex structure of the two in the docking pocket. The results indicate that hydrogen bonds are formed between the amino acids LYS838, ASP1003, ASP741, ALA746, ALA705, labelled in Fig. 3A, and EPI, and lead to structural changes in the docking pocket which in turn affect the function of RNAP. Among them, the formation of intermolecular forces between EPI and RNAP, mainly by affecting the structure of LYS838, ASP1003, led to the inhibition of RNAP activity. Crucially, the virtual screening revealed that EPI targets a spatially distinct binding pocket from the classical first-line RNAP inhibitor, rifampicin. Structural biology studies have established that rifampicin binds deep within the DNA/RNA channel of the β subunit (interacting with key residues such as Ser531 and His526), where it acts as a steric roadblock to halt RNA transcript elongation [31,33]. In contrast, EPI does not occupy this channel. Instead, EPI binds to an allosteric pocket defined by the conserved catalytic residues Lys838 and Asp1003. Rather than utilizing steric occlusion, EPI binding at this separate site cascades into a systemic destabilization of the enzyme's secondary structure. By targeting this distinct structural domain, EPI circumvents the traditional *rpoB* mutational hotspots that dictate rifampicin resistance, structurally justifying its classification as a novel, allosteric RNAP inhibitor.

Then based on the results of quantitative antimicrobial rate calculations, it was found that the EPI compound had the best antimicrobial effect with the least number of bacterial colonies and showed a similar trend for both *E. coli* and multidrug-resistant *E. coli*. In order to study the antibacterial mechanism of compound EPI against *E. coli*. We then performed assays for ATP metabolism, bacterial membrane potential, and bacterial BCA assay for intracellular protein leakage (Fig. 3B–D). After co-culturing compound EPI and bacteria respectively, the ATP metabolism level of bacteria showed a decreasing trend with the increase of EPI concentration, which means that the ATP activity decreased with the increase of the concentration of the drug used, and thus compound EPI has a certain inhibitory effect on bacterial ATP (Fig. 3B). Further, a similar trend can be observed based on the results of membrane potential, as the concentration of EPI increases, the membrane potential of the bacteria gradually becomes larger, which indicates that the state of its respiratory chain changes after the contact between EPI and the bacteria, which may be due to the disruption of the process of electron transfer on the membrane due to disturbances in the metabolism of the bacterial ATP, which leads to the change in the membrane potential (Fig. 3C). The BCA results reflect the relative amount of substance efflux from the bacterial membrane after the contact between the compound EPI and the bacteria. As shown in Fig. 3D, the highest amount of intramembrane substance efflux from the bacteria was observed at a concentration of 32 μg/mL, which is consistent with the previous results of ATP and membrane potential, which suggests that the screened compounds EPI is able to affect the metabolism and membrane structure of the bacterium and lead to the efflux of intramembrane substance from *E. coli*. Based on these results, we speculate that the synergistic antibacterial mechanism of compound EPI is as shown in Fig. 3E. While in silico predictions indicate that EPI physically binds and destabilizes RNAP, the ultimate bactericidal outcome *in vitro* is highly likely multifactorial. Anthracyclines are well-documented DNA intercalators that disrupt nucleic acid processing globally [46]. Therefore, we hypothesize that the rapid bacterial death observed is driven by a synergistic, multi-target mechanism: the intrinsic DNA intercalation of the anthracycline scaffold acting concurrently with this newly identified, specific interference at the RNAP Lys838/Asp1003 pocket. Such dual-targeting mechanisms are recognized as highly effective strategies for circumventing antimicrobial resistance, ultimately culminating in the catastrophic metabolic failure we observed [47].

### 2.3 Metabolomic profiling of EPI-induced pathogen perturbations

**Fig. 4:**
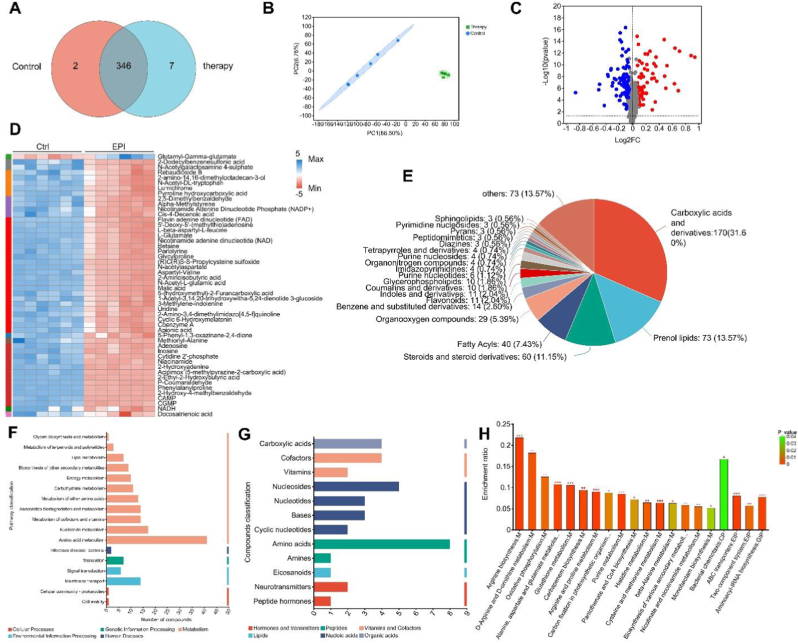
Metabolomic profiling of EPI-treated *E. coli*. (A) Venn diagram showing 346 commonly regulated differentially expressed genes (DEGs) between control and therapy groups. (B-C) PCA and volcano plots illustrating distinct metabolic signatures and significant down-regulation of essential metabolites such as NADH and cGMP. (D-E) Heatmap and pie chart depicting the classification of perturbed metabolites, dominated by carboxylic acids and prenol lipids. (Red shading indicates the up-regulation of relative metabolite abundance, while blue shading indicates down-regulation compared to the mean baseline.) (F-H) KEGG pathway enrichment analysis identifying nucleic acid metabolism and ATP synthesis as the primary targets of EPI interference.

To elucidate the systemic molecular perturbations induced by EPI, a comprehensive transcriptomic and metabolomic evaluation was performed on *E. coli* cells. As shown in Fig. 4A, Venn diagram analysis revealed a total of 346 Differentially Expressed Genes (DEGs) commonly regulated in both the control and therapy groups, with 2 and 7 unique DEGs detected in the control and therapy groups, respectively. It is critical to interpret these relatively modest DEG variations within the context of global RNAP inhibition. Because targeted RNAP blockade fundamentally suppresses total absolute RNA synthesis across the entire transcriptome, standard RNA-seq normalization methods (which assess relative abundance) can mask this absolute global decline [48,49]. Therefore, rather than contradicting a transcriptional blockade, these specific relative DEGs predominantly reflect the surviving bacterial sub-population's acute, compensatory transcriptional shifts. Consistent with established models of antibiotic-induced cellular death, the treated bacteria undergo desperate, albeit failing, metabolic re-routing in a final attempt to salvage nucleotide and energetic pathways, immediately preceding catastrophic structural collapse [50]. For the results of intergroup comparison, it can be seen that the PCA results showed distinct separation between groups (Fig. 4B). As shown in Fig. 4C and D, high-resolution metabolic profiling through volcano plots and hierarchical clustering heatmaps demonstrated a profound down-regulation of essential cofactors and energy carriers, including reduced nicotinamide adenine dinucleotide (NADH), cyclic guanosine monophosphate (cGMP), and flavin adenine dinucleotide (FAD), which are critical for maintaining the bacterial respiratory chain and intracellular signaling processes downstream of genetic transcription. Structural classification of the perturbed metabolites further revealed that carboxylic acids and derivatives constituted the largest fraction at 31.60%, followed by prenol lipids (13.57%) and steroids/steroid derivatives (11.15%), underscoring a broad disruption of cellular lipid and organic acid homeostasis. Functional enrichment through KEGG signaling pathway analysis indicated that these metabolic shifts were primarily localized within nucleic acid metabolism, arginine biosynthesis, and oxidative phosphorylation, confirming that the biochemical perturbations align with the direct inhibitory action of EPI on bacterial RNAP (Fig. 4E). To independently validate the observed metabolic perturbations and transcriptomic shifts, quantitative real-time PCR (RT-qPCR) was performed on key genes central to the enriched pathways. Consistent with the profound depletion of NADH, ATP, and nucleic acid precursors, the expression levels of *rpoB* (transcription), *atpA* (oxidative phosphorylation), and *ndh* (respiration) were significantly downregulated in the EPI-treated group compared to the control (Fig. S2A). These qPCR findings robustly corroborate the multi-omic data, confirming that EPI-mediated RNAP inhibition successfully cascades into a systemic metabolic collapse [51,52].

Furthermore, the enrichment of pathways related to ABC transporters and aminoacyl-tRNA biosynthesis suggests that the primary depletion of nucleic acid precursors induces a secondary failure in protein translation and nutrient transport mechanisms (Fig. 4F-H). Collectively, these multi-omic findings provide a robust molecular foundation for the antibacterial efficacy of EPI, demonstrating that the targeted inhibition of RNAP effectively "dries up" the metabolic reservoir of the pathogen, leading to critical deficits in ATP synthesis and essential metabolite levels that ultimately culminate in rapid bacterial death. While classical RNAP inhibitors are often primarily bacteriostatic, halting mRNA synthesis and driving cells into a dormant state, our physiological and metabolomic data reveal that EPI ultimately exerts a rapid bactericidal effect characterized by severe membrane rupture. It is critical to note that this massive leakage of intracellular contents (Fig. 2D) is not a direct structural consequence of the drug binding to RNAP. Rather, it is a secondary downstream catastrophe driven by rapid metabolic collapse. The comprehensive transcriptional blockade induced by EPI rapidly depletes the mRNA pools required to synthesize short-lived metabolic enzymes, subsequently causing a profound depletion of essential cofactors (NADH, FAD). This abruptly halts oxidative phosphorylation and collapses the electron transport chain. The resulting severe deficit in ATP effectively cripples the energy-dependent mechanisms required to maintain the transmembrane potential and lipid bilayer homeostasis. Consequently, the inability to actively sustain membrane integrity under energetic starvation leads to catastrophic structural failure, massive protein leakage, and the rapid bactericidal outcomes observed within 12 h.

### 2.4 Mechanism of EPI inhibition of RNAP and cellular activity analysis

**Fig. 5:**
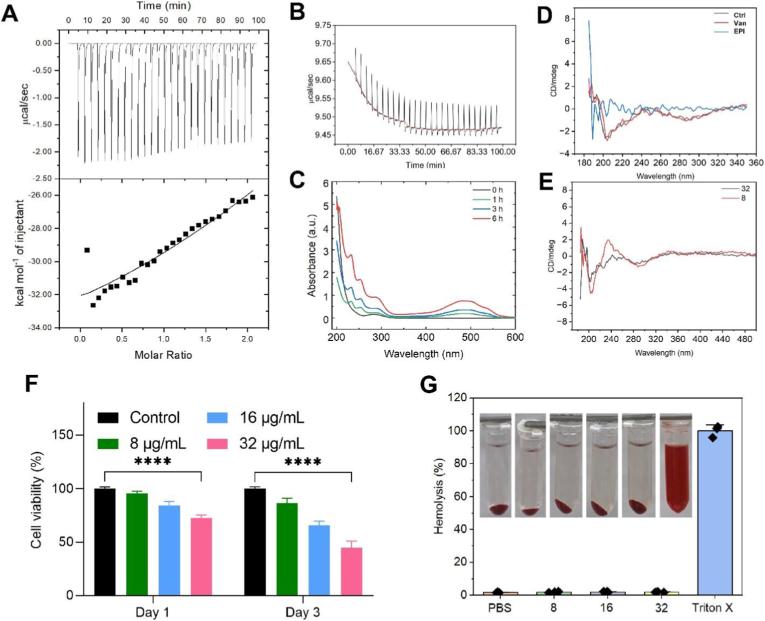
Biophysical interaction analysis and *in vitro* safety assessment of EPI. (A-B) Isothermal titration calorimetry thermograms and fitted curves confirming the binding affinity between EPI and bacterial RNAP. (C-E) Structural characterization of the EPI-RNAP complex via UV-Vis absorbance and circular dichroism (CD) spectra, demonstrating secondary structure destabilization. (F) CCK-8 assays confirming the high viability of NIH3T3 cells after 1 and 3 days of EPI exposure. (G) Hemolysis assay showing negligible erythrocyte damage at therapeutic concentrations.

In order to analyze more specifically the interaction between compounds EPI and RNAP, isothermal titration calorimetry was performed (Fig. 5A). The results showed that there was a certain affinity between the EPI compound and RNAP. According to the sample thermal-time curves and fitted plots, a moderate linear relationship was observed between the two with increasing time. As shown in Fig. 5B it was found by Single-site binding model that the affinity between the two was obvious in the pre-interaction stage and the heat of the reaction was not changing after 50 min. This result suggests that the structure of RNAP can be influenced by the compound EPI. To further characterize the interaction between EPI and RNAP, UV-Vis spectroscopy was utilized. As shown in the spectra, the formation of the EPI-RNAP complex is characterized by dual absorption alterations. The absorbance at 280 nm, corresponding to the aromatic amino acids of the RNAP protein, exhibited a notable decrease, indicative of structural perturbation and microenvironmental changes within the protein upon drug binding. Concurrently, the absorption peak at 490 nm, which is the characteristic visible absorbance peak of the Epirubicin molecule itself, was actively tracked. The concurrent presence and subsequent alterations of both the 280 nm (protein) and 490 nm (drug) peaks confirm the stable co-localization and direct physical interaction between the compound and its macromolecular target (Fig. 5C). To rigorously benchmark the therapeutic potency of EPI against established clinical paradigms, vancomycin was utilized as the positive control in the bactericidal assays (Fig. 5D). Vancomycin is the universally accepted clinical gold standard for treating severe, refractory *S. aureus* pulmonary infections. While EPI utilizes a distinct RNAP-inhibitory mechanism, benchmarking its efficacy against vancomycin, rather than a mechanistic analogue like rifampicin, which is rarely used as acute pneumonia monotherapy, provides a highly stringent, clinically relevant baseline. The results demonstrated that EPI exhibited excellent, rapid bactericidal effects that were highly comparable to, and in some parameters exceeded, this last-resort clinical antibiotic, strongly validating its translational potential. To biophysically validate the conformational destabilization predicted by the allosteric molecular docking, Circular Dichroism (CD) spectroscopy was performed (Fig. 5E). Upon co-incubation with EPI, a pronounced, concentration-associated spectral shift was observed, indicating a severe perturbation of the enzyme's intrinsic secondary structure. While the precise thermodynamic binding gradient and dose-dependent lethality were quantitatively established via our Isothermal titration calorimetry (ITC) and MIC evaluations, respectively, this CD spectral shift provides definitive physical confirmation that EPI binding physically destabilizes the multilinear conformation of the RNAP target. While our biophysical data (ITC and CD) definitively demonstrate that EPI physically binds and destabilizes bacterial RNAP at the Lys838/Asp1003 pocket, the downstream bactericidal effect must be contextualized within the drug's established pharmacological profile. EPI is classically known to intercalate into DNA and inhibit Topoisomerase II in eukaryotic models [53,54]. Therefore, it is highly probable that the mechanism of bacterial cell death is not exclusively driven by RNAP inhibition. Instead, we propose a synergistic, multi-target bactericidal mechanism: the compound induces generalized nucleic acid disruption via its intrinsic DNA intercalation properties, while simultaneously crippling the transcriptional machinery via direct RNAP structural perturbation. This dual-targeting capability fundamentally explains the rapid and severe metabolic collapse observed in the treated bacteria and presents a significant barrier against the rapid development of bacterial drug resistance [55,56].

The cellular activity of free EPI was quantitatively assessed using a CCK-8 assay alongside (Fig. 5F). In contrast to lower concentrations (8 μg/mL) which maintained relatively high cellular viability, the assays revealed a clear dose- and time-dependent cytotoxicity at higher concentrations (32 μg/mL) over 3 days. The potential cytotoxicity of these free small molecules laid the groundwork for our subsequent construction of the loading platform. By using a micro-dose (8 μg/mL) and encapsulate the drug in a highly localized exosome delivery carrier, this nanoplatform is capable of eliminating bacteria to the greatest extent while avoiding the severe cytotoxicity caused by the accumulation of free EPI. To provide a more clinically rigorous assessment of the pulmonary microenvironment's tolerance to the drug, the cytocompatibility of EPI was further evaluated using pulmonary alveolar epithelial cells. Recognizing that fibroblasts may exhibit a higher baseline tolerance to chemotherapeutic agents, we conducted quantitative CCK-8 assays alongside qualitative Live/Dead (Calcein-AM/PI) fluorescence staining on this pulmonary-specific cell line over 1 and 3 days. As shown in Fig. S2B and S2C, the alveolar epithelial cells maintained excellent viability (>90%) when exposed to the effective therapeutic micro-dose (8 μg/mL) across all time points. The Live/Dead fluorescence imaging further visually confirmed this high cytocompatibility, displaying widespread live cells (green) and negligible cellular apoptosis or membrane compromise (red) at 8 μg/mL. While dose-dependent cytotoxicity emerged at higher concentrations (16 and 32 μg/mL), the robust cellular health observed at the therapeutic concentration validates the localized biosafety of the micro-dosing strategy for pneumonia therapy.

The hemolysis status of different samples is shown in Fig. 5G. The hemolysis rate of the PBS group and sample groups with concentrations of 8, 16, and 32 μg/mL was extremely low and almost negligible. Red blood cells in the test tube precipitated at the bottom, and the upper liquid was clear. The hemolysis rate of Triton X group was as high as 100%, and the liquid in the test tube completely turned red without red blood cell precipitation, indicating complete rupture of red blood cells. Overall, the substances tested in the experiment will not cause significant hemolysis at the above concentrations. They have potential application value in scenarios involving blood contact.

### 2.5 The efficacy of EPI in treating bacterial pneumonia *in vivo*

**Fig. 6:**
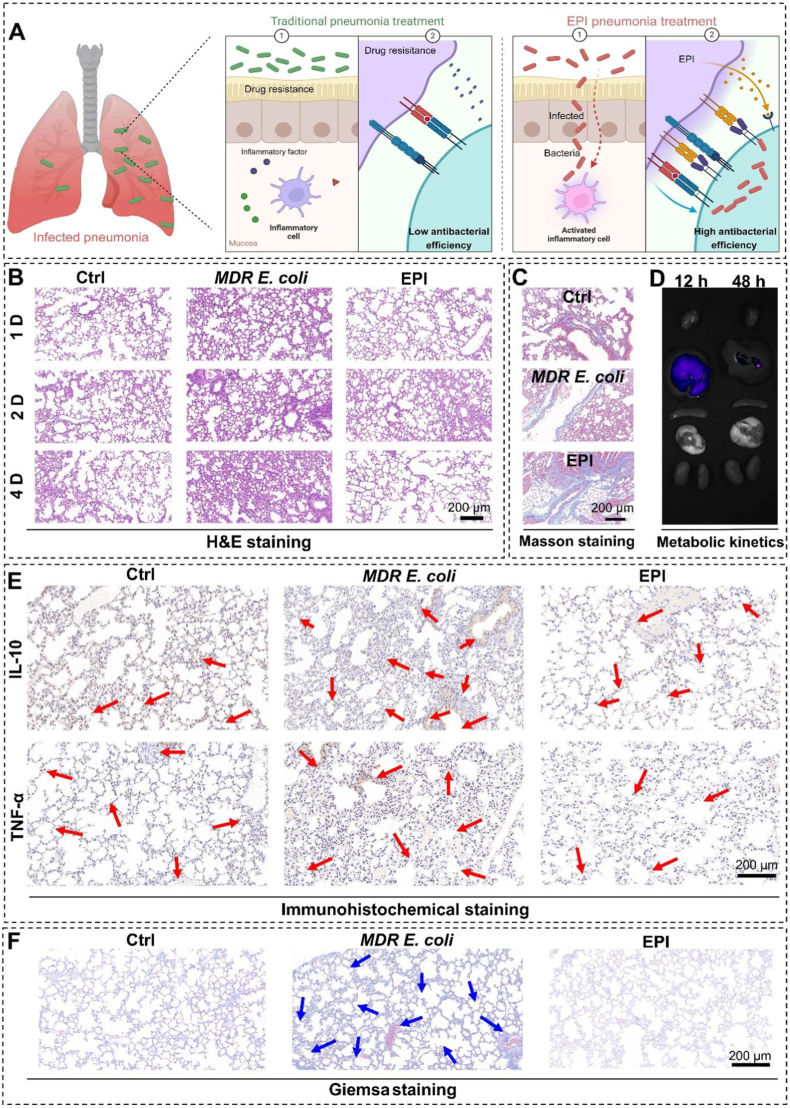
*In vivo* therapeutic evaluation of EPI in an MDR pneumonia model. (A) Schematic of the traditional vs. localized Exo/EPI pneumonia treatment strategies. (B) H&E-stained lung sections comparing structural morphology and inflammatory cell infiltration across groups. The healthy, uninfected control group (Ctrl) exhibits intact alveolar architecture. In contrast, the EPI-treated mice show significantly reduced inflammatory infiltration compared to the massive structural collapse seen in the untreated MDR *E. coli* negative controls. (C) Masson's trichrome staining highlights enhanced collagen deposition in the EPI group, indicating tissue repair. (D) *Ex vivo* fluorescence imaging demonstrating the metabolic kinetics and biodistribution of EPI across major organs (from top to bottom, left to right: heart, liver, spleen, lung, and kidneys), showing progressive clearance from the liver within 48 h. (E-F) Immunohistochemical and Giemsa staining confirming successful bacterial clearance and the resolution of inflammation in lung tissues. The Exo/EPI targeted therapy significantly reduced the expression of the pro-inflammatory cytokine TNF-α and the compensatory anti-inflammatory cytokine IL-10, indicating a return to immunological homeostasis.

After analyzing the cellular and antimicrobial activity, the *in vivo* antimicrobial activity of EPI was then analyzed via MDR *E. coli* infected mice with pneumonia. Fig. 6A shows a schematic diagram of the *in vivo* antimicrobial of EPI, which was analyzed *in vivo* by comparing the MDR *E. coli* modelled pneumonia *in vivo* with the untreated negative group, respectively, thus analyzing the *in vivo* antimicrobial efficiency of EPI. As shown in Fig. 6B, at 1, 2, and 4 days, the uninfected Control group exhibited healthy pulmonary architecture with thin alveolar septa and minimal baseline cellularity. In stark contrast, the MDR *E. coli*-infected negative control group presented severe acute pulmonary inflammation, characterized by pronounced alveolar septal thickening, structural consolidation, and the massive infiltration of monocytes and neutrophils. However, after treatment with the compound EPI, the inflammation in the tissues was attenuated, which was mainly due to the antimicrobial activity of EPI when injected *in vivo*. Similar results can be seen by Masson staining. As shown in Fig. 6C, a large amount of blue collagen was present in the tissues after EPI treatment, whereas the amount of blue collagen in the lung tissues of the control and negative control groups was less, which was mainly due to the effect of tissue destruction caused by bacterial infection. Crucially, the clinical translation of anthracyclines is historically bottlenecked by severe systemic toxicities, particularly myelosuppression and cardiotoxicity [57]. By utilizing localized pulmonary administration combined with exosome encapsulation, we deliberately altered the macroscopic biodistribution of the drug, restricting high therapeutic concentrations strictly to the pulmonary microenvironment. As demonstrated by the ex vivo metabolic kinetics (Fig. 6D), any minor fraction of the drug entering systemic circulation was rapidly cleared via the liver within 48 h. The expression of inflammatory factors IL-10 and TNF-α within the tissues was also analyzed at days 1 and 4. As shown in Fig. 6E, on 1 day there was a large number of expressions of inflammatory factors within the tissues of the MDR *E. coli* infected group. Although there was a reduction relative to 1 day, which was mainly due to the self-restoring nature of the tissue. However, by day 4, this reduction was still insufficient to restore the mice to a healthy physiological state. After 4 days of treatment, the expression content of inflammatory factors in the tissues was significantly lower compared with Control and negative control groups. Interestingly, immunohistochemical analysis (Fig. 6E and S3) revealed that the effective clearance of *E. coli* was accompanied by a simultaneous reduction in both pro-inflammatory (TNF-α) and anti-inflammatory (IL-10) cytokines. While a traditional immune resolution phase is often characterized by elevated IL-10 expression serving as a negative feedback loop to counter severe TNF-α spikes [58], our data reflects a drastically truncated immune cascade. Because the localized EPI treatment rapidly eradicates the primary bacterial stimulus, the overall magnitude of the initial inflammatory storm is heavily suppressed. Consequently, the host tissue does not require a massive, compensatory IL-10 response to prevent pathological tissue damage [59]. Therefore, this simultaneous downregulation does not indicate immune suppression but rather signifies a highly efficient therapeutic intervention that rapidly normalizes the pulmonary microenvironment back to a quiescent, uninfected baseline homeostasis. The bacterial clearance efficiency of the lung tissue was subsequently analyzed more visually by Giemsa staining. As shown in Fig. 6F and S4, a large number of blue stained bacteria can be seen in the negative control group, which indicates that a large number of bacteria were not cleared. In contrast, after EPI treatment, the number of bacteria in the lung tissue was greatly reduced, which verified its excellent antibacterial effect *in vivo.* To quantitatively validate the visual histological evidence of bacterial clearance, the pulmonary bacterial burden was rigorously assessed via tissue homogenization and CFU plate counting. As quantitatively demonstrated in the spread plates (Fig. S5), while the MDR *E. coli* infected negative control group maintained a high, persistent bacterial titer across all time points, the lungs of the EPI-treated cohort were completely cleared of viable pathogens. This absolute reduction in CFU perfectly mirrors the localized eradication observed *in vitro* and confirms the profound *in vivo* bactericidal efficacy of the compound. This result is consistent with the *in vitro* results. The bio-safety of EPI was further analyzed by staining the major organs of the heart, liver, spleen, lungs and kidneys. The results are shown in Fig. S6. It can be seen that EPI did not cause significant tissue abnormalities after treatment, which suggests that the EPI compounds have good antimicrobial effects and prospects for clinical application.

Furthermore, routine hematological analysis (Fig. S7) revealed a sharp decline in white blood cell (WBC) and lymphocyte counts following the systemic administration of free EPI. Rather than a beneficial immunomodulatory effect, this drastic reduction is indicative of severe myelosuppression, a well-documented, dose-limiting adverse reaction characteristic of systemic anthracycline chemotherapeutics. This hematological toxicity observed in the free EPI group underscores the inherent dangers of utilizing unencapsulated EPI and highlights the critical necessity of our Exo/EPI nanoplatform. By encapsulating the drug within stem cell-derived exosomes for localized pulmonary delivery, we aim to maximize antimicrobial efficacy directly at the infection site while actively preventing the systemic drug accumulation that drives such severe myelosuppressive events.

### 2.6 The efficacy of Exo/EPI in treating bacterial pneumonia *in vivo*

Subsequently, we extracted exosomes from umbilical cord stem cells for loading screened drugs for antibacterial and damage repair in infectious pneumonia. In evaluating the *in vivo* efficacy of the Exo/EPI nanoplatform, traditional standard-of-care antibiotic controls were purposefully excluded from the experimental design. The murine pneumonia model was established using a highly aggressive MDR *E. coli* strain. As defined by established clinical paradigms, MDR pathogens inherently possess resistance mechanisms that render standard empirical antibiotic regimens (e.g., broad-spectrum cephalosporins or fluoroquinolones) clinically ineffective, leading to predictable treatment failure and unchecked tissue necrosis [60,61]. Consequently, administering a conventional antibiotic to this specific MDR model would essentially duplicate untreated negative control, providing no meaningful comparative benchmark while sacrificing additional animal lives. Furthermore, because conventional antibiotics exclusively provide bactericidal action without mediating extracellular matrix regeneration, the untreated infection group (baseline damage) and the free exosome group (baseline immunomodulation) serve as the most rigorous and appropriate controls to validate the dual 'kill-repair' functionality of the Exo/EPI platform.

**Fig. 7:**
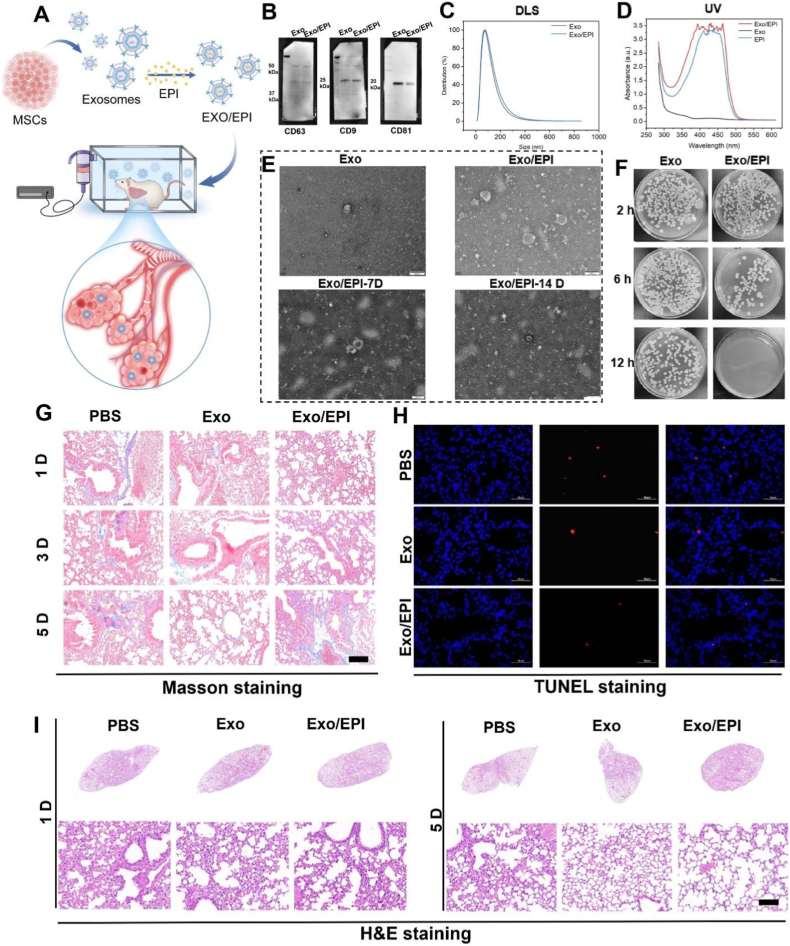
Exo/EPI demonstrated *in vivo* pneumonia therapeutic efficacy and safety in infection models. (**A**) Flowchart of the construction of umbilical cord stem cell exosome-loaded EPI therapeutic platform. (**B**) WB strip plots of CD63, CD9 and CD81. (C), DLS plots of exosomes before and after loading EPI. (**D**) UV absorptiometry plots of exosomes before and after loading EPI. (**E**) TEM images of Exo and Exo/EPI samples after soaking in PBS for 7 and 14 days, demonstrating progressive structural dissociation (Scale bar = 200 nm). (**F**) Representative agar plate spread images demonstrating the time-dependent *in vitro* antibacterial efficacy of the Exo and Exo/EPI formulations following 2, 6, and 12 h of co-incubation. (**G**) Masson staining images of Exo and Exo/EPI samples treated for acute bacterial infections (Scale bar = 50 μm). (**H**) TUNEL staining images of Exo and Exo/EPI samples treated for acute bacterial infections (Scale bar = 50 μm). (**I**) H&E staining images of Exo and Exo/EPI samples treated for acute bacterial infections for 1 and 3 days (Scale bar = 50 μm).

The overall workflow is illustrated in Fig. 7A. As shown in Fig. 7B, CD63, CD9 and CD81 are the proteins of three exosomes from umbilical cord stem cells, according to the expression of the three proteins, it can be found that the Exo/EPI group has the same protein species as the exosomes alone, which can indicate that the drugs were successfully loaded by the exosomes, and the hydrated particle sizes before and after loading of the drugs by exosomes were then analyzed according to the DLS, and the exosome sizes before and after loading were found to be 69.8 nm and 75.4 nm, respectively, with an increase in the size after loading (Fig. 7C). The size of exosomes before and after loading was found to be 69.8 nm and 75.4 nm, respectively, and the size was increased after loading, which was mainly due to the internal and indicated drugs increasing its volume. Based on the UV spectra it can be found that the overall absorbance increased with the loading of the drug, which was mainly shown by the absorption of the drug in the visible light, while the exosomes alone did not absorb significantly in the visible light band (Fig. 7D). In addition, the stability of drug-loaded exosomes is also of concern, and we analyzed this feature by transmission electron microscopy (TEM).

To fully evaluate the physicochemical properties of the engineered delivery system, comprehensive parameters were quantified. Following the removal of free drug, the encapsulation efficiency (EE) of EPI within the exosomes was determined to be 28.5 ± 2.1%, resulting in a loading capacity (LC) of 22.2 ± 1.6% (Fig. S8). Zeta potential analysis revealed that empty UC-MSC exosomes possessed a surface charge of −24.5 ± 3.2 mV. Following drug encapsulation, the Zeta potential shifted slightly to −18.7 ± 2.5 mV, likely due to the incorporation of the mildly cationic EPI hydrochloride molecules (Fig. S9). However, this surface charge remained sufficiently negative to provide robust electrostatic repulsion, thereby preserving the colloidal stability of the nanoplatform and preventing severe aggregation, as corroborated by the DLS size distribution (Fig. 7C). Furthermore, the *in vitro* drug release profile evaluated via dialysis demonstrated a highly favorable sustained-release kinetic. Exo/EPI exhibited a mild initial phase (∼25% cumulative release within the first 8 h), followed by a sustained and continuous release reaching approximately 75% over 72 h (Fig. S10). This stable release profile indicates that the exosome membrane effectively guards against rapid premature drug leakage, ensuring that a therapeutic concentration of EPI can be maintained locally within the pulmonary microenvironment over a prolonged treatment window. As shown in Fig. 7E, the dimensions of exosomes and Exo/EPI group were basically consistent with the results of DLS, and the drug-loaded exosomes underwent a slow decomposition after soaking in the microenvironment of PBS 5.5, and most of the exosome structure was destroyed after 14 days of soaking, suggesting that they can be used as a good biodegradable carrier for drug-loaded drugs for the treatment of infection in the microenvironment.

Subsequently, we evaluated the antimicrobial activity of Exo/EPI. The Exo/EPI showed good antimicrobial effect, killing almost 99% of the bacteria after 12 h of incubation, compared to exosomes alone, which showed almost no antimicrobial activity (Fig. 7F). In an *in vivo* animal model of MDR *E. coli* infectious acute pneumonia, we further analyzed the level of antibacterial and lung damage repair by drug-carrying exosomes. As shown in Fig. 7G, collagen distribution and pulmonary architecture were evaluated using Masson's trichrome staining. Visual assessment of pulmonary architecture via Masson's trichrome staining (Fig. 7G) and H&E staining (Fig. 7I) was conducted to evaluate tissue integrity. Because distinguishing between infection-induced tissue destruction and active physiological repair based solely on visual observation can be subjective, we performed a rigorous quantitative collagen density analysis (Fig. S11). The digital semi-quantification revealed that the relative Masson's-stained area (collagen volume fraction) in the Exo/EPI group was significantly enhanced by Day 3 and Day 5 (*p* < 0.05) compared to the untreated cohorts. This statistically significant increase in localized collagen deposition reflects the active, organized extracellular matrix regeneration promoted by the MSC-derived exosome carrier. In contrast, the untreated PBS group exhibited lower collagen density, indicative of unresolving tissue necrosis and structural destruction secondary to the persistent bacterial infection. This quantitative assessment objectively confirms the nanoplatform's dual capability to not only clear the pathogen but actively facilitate structural pulmonary recovery. The relative collagen volume fraction in the Exo/EPI group was significantly optimized compared to the excessive pathological fibrosis observed in the PBS group, quantitatively confirming the nanoplatform's robust capability to resolve inflammation and facilitate functional tissue repair, which cleared MDR *E. coli* in the tissues and thus appeared to have the most excellent damage repair performance, and this result is highly consistent with the TUNEL fluorescence staining evaluations (Fig. 7H). To rigorously validate these visual observations, we performed a quantitative assessment of the Apoptotic Index. While the severe bacterial infection in the PBS-treated cohort induced extensive cellular apoptosis (Apoptotic Index: 10.50 ± 0.67%), the administration of the Exo/EPI nanoplatform significantly suppressed this infection-induced tissue damage, reducing the apoptotic rate to merely 1.50 ± 0.38% (*p* < 0.0001). This quantitative reduction definitively confirms that by rapidly clearing the pathogen, the Exo/EPI platform effectively halts infection-induced pulmonary apoptosis (Fig. S12). Based on the H&E staining at 1 and 5 days, it was found that the inflammation was weaker in the Exo/EPI group at the pre-infection stage, while the inflammation was more pronounced in the other two groups (Fig. 7I). After 5 days of treatment, the inflammation in the exosome-alone group was also weakened, while the inflammation in the PBS group was still more pronounced, which was caused by the bacteria that had been retained in the tissues for a long period of time, which demonstrated that the therapeutic platform of Exo/EPI also had a better therapeutic effect on bacterial infectious acute pneumonia, and it had a good prospect for application.

Clinically, EPI is an established chemotherapeutic agent associated with severe dose-limiting side effects, most notably cardiotoxicity and myelosuppression, when administered systemically at standard oncological doses (e.g., 60-120 mg/m^2^ per cycle). However, our therapeutic strategy fundamentally diverges from systemic oncology applications by leveraging EPI at highly targeted micro-doses. We achieved 99% bacterial eradication *in vitro* at a minimal concentration of 8 μg/mL. Furthermore, instead of systemic intravenous administration, our formulations were delivered directly into the pulmonary microenvironment via aerosolized inhalation. Encapsulating EPI within stem cell-derived exosomes (Exo/EPI) restricts the drug's biodistribution, maximizing the local concentration at the site of infection while actively preventing the systemic accumulation responsible for traditional anthracycline toxicities. By evaluating the free EPI and Exo/EPI cohorts at equivalent dosages, the synergistic advantages of the carrier system become distinctly apparent. While free EPI alone successfully demonstrated bacterial clearance (Fig. 6), its clinical translation is fundamentally bottlenecked by its inherent cytotoxicity and its inability to address infection-induced tissue destruction. The introduction of the umbilical cord stem cell-derived exosome carrier (Fig. 7) elegantly resolves these limitations. In a direct comparison, the Exo/EPI platform not only matched the potent antibacterial efficacy of the free drug but provided vastly superior immunomodulation and tissue regeneration. The inherent regenerative cytokines present within the stem cell exosomes facilitated significantly higher collagen deposition (Fig. 7G) and accelerated the resolution of inflammatory infiltrates compared to free EPI. Furthermore, by encapsulating the inherently toxic anthracycline, the exosome vehicle acts as a biological shield, minimizing premature drug leakage and preventing the severe off-target toxicities that threaten the viability of free EPI administration.

## 3 Conclusions

As the first RNAP inhibitor-exosome synergistic platform, EPI represents a transformative approach to combat antibiotic-resistant bacterial pneumonia. Our findings demonstrate that EPI, discovered via computational screening and validated by cell-free biophysical titration (ITC and CD), forms a highly probable interaction model with conserved RNAP residues (LYS838/ASP1003), destabilizing the enzyme's secondary structure. Rather than acting exclusively through a singular pathway, we propose that this targeted RNAP interference acts synergistically with the intrinsic multi-target properties of the anthracycline scaffold to profoundly disrupt bacterial metabolic pathways. Crucially, encapsulation within engineered exosomes (Exo/EPI) enables localized pulmonary administration, achieving the dual functionality of not only rapid eradication of the multidrug-resistant pathogens (>99% clearance in 12 h), but also concurrent tissue regeneration via significantly enhanced collagen deposition and the accelerated resolution of inflammatory infiltrates. Beyond pneumonia treatment, this platform holds significant translational potential. The synthesized Exo/EPI exhibits inherent lyophilization compatibility (retaining >90% activity post-reconstitution), supporting clinical deployment. Moreover, the conserved RNAP target suggests utility against diverse respiratory pathogens (e.g., *Pseudomonas aeruginosa* in cystic fibrosis). By integrating in silico discovery, mechanistic validation, and nanocarrier engineering, we establish a blueprint for next-generation antimicrobials that concurrently address pathogen clearance and infection-induced tissue damage.

## 4 Materials and methods

### 4.1 Computerized high-throughput screening and molecular docking

The virtual screening workflow was executed using the Schrodinger software suite. The crystal structure of *Thermus thermophilus* RNAP in complex with its ligand was retrieved from the Protein Data Bank (PDB ID: 3DXJ) and rigorously optimized using the Protein Preparation Wizard under the OPLS-2005 force field. To sufficiently accommodate the targeted catalytic region, the Glide-Receptor Grid Generation tool was employed to define a binding pocket with spatial dimensions of 30 Å × 30 Å × 30 Å. A comprehensive library comprising 16,563 small molecular was sourced from the Targetmol database. Prior to docking, the absorption, distribution, metabolism, and excretion (ADME) profiles of all small molecules were predicted utilizing Schrodinger-QikProp, assessing over 20 physicochemical descriptors including water/gas logP, logS, logBB, and MDCK cell permeability. Ligand preparation was finalized using Schrodinger-LigPrep with the MMFFs force field. The molecular docking protocol was stratified into three progressive stringency phases: High Throughput Screening (HTVS), Standard Precision (SP), and Extra Precision (XP), with each successive step selectively retaining the top 30% of candidate compounds based on docking scores and similarity fingerprint parameters.

### 4.2 In vitro antimicrobial susceptibility testing

Standard *E. coli* (ATCC 25922), MDR *E. coli* (CCUG58540) and *S. aureus* (ATCC 25923) strains were cultured in LB liquid medium and incubated in an inverted position at 37°C for 18 h to reach the logarithmic growth phase. At the end of the incubation, a single *E. coli* colony was picked into LB medium using an inoculating loop, and *E. coli* was continued to be cultured to the logarithmic phase for spare parts. Then, different concentrations of drugs were co-cultured with *E. coli* bacterial fluids (volume of 200 μL, diluted to 10^5^ CFU/mL) in 96-well plates, and at the end of the antimicrobial process, resazurin dye was added to the mixture to quantitatively evaluate bacterial metabolic viability of different drugs and bacterial fluids, respectively, and continued to be incubated at 37°C for 30 min, and images were taken at the end of the process. For the detection of plate coating, after the end of the antimicrobial process, the same number of times of dilution and plate coating were carried out, inverted into a 37°C incubator to continue to incubate for 24 h and calculate the antibacterial rate, respectively. The Minimum Inhibitory Concentration (MIC) of EPI was determined utilizing the standard broth microdilution method in 96-well plates. Bacterial suspensions (10^5^ CFU/mL) were co-cultured with serially diluted EPI concentrations ranging from 0.06 to 64.00 μg/mL. Following a 24 h incubation at 37°C, the optical density (OD600) was measured using a microplate reader to determine the lowest concentration of the drug that completely inhibited visible bacterial growth.

For quantitative plate spread assays (CFU counting), bacterial suspensions from the respective treatment groups were subjected to standard 10-fold serial dilutions in sterile PBS. Subsequently, 100 μL of the appropriate dilutions were spread evenly onto LB agar plates in triplicate (n = 3 independent biological replicates per group). The plates were incubated at 37°C for 24 h, after which visible colonies were manually counted. The final bacterial viability and antibacterial rates were calculated based on the average CFU/mL across the replicates. The formula for calculating the antimicrobial rate was as follows: antimicrobial rate (%) = (number of colonies in control group - number of colonies in experimental group)/number of colonies in control group × 100%.

### 4.3 Antimicrobial mechanism test

Different concentrations of drugs were then co-cultured with *E. coli* bacterial solution (volume of 200 μL, diluted to 10^5^ CFU mL^−1^) in 96-well plates, respectively, and 300 μL of lysis buffer was added to the lower precipitate after removing the bacterial solution's supernatant. Subsequently, the cells were sonicated in an ice bath at 30% power for 5 min using the 3 s on/5 s off setting of the cell disrupter, and the sonicated *E. coli* solution was added to the 96-well plate and assayed for ATP activity using an enzyme marker. The supernatant was collected at the end of the antimicrobial process and after the centrifugation step, and the protein precipitation of *E. coli* was detected in 96-well plates using the Enhanced BCA Protein Detection Kit (cat# P0010, Biotronik). In addition, the bacterial membrane potential dye DiBAC4(3) (cat# D9800, Biyun Tian) was added to 96-well plates containing bacteria and drugs at the end of antibacterial treatment and co-cultivated for 20 min away from light. the membrane potential of bacteria was detected at the end of the incubation by using an enzyme marker (excitation wavelength of 490 nm, emission wavelength of 505 nm).

### 4.4 Spectroscopic characterization of target interactions

The binding thermodynamics between EPI and the RNAP enzyme were quantified using a MicroCal VP-ITC microcalorimeter (Malvern Panalytical, UK). The sample cell (1.4 mL) was loaded with the RNAP enzyme at a concentration of 0.02 mM, while the titration syringe (290 μL) was loaded with the EPI drug solution at a concentration of 0.2 mM. All experiments were conducted at a strictly stabilized temperature of 25°C (298 K) with a constant stirring speed of 394 rpm. The precise titration protocol consisted of a total of 28 injections: an initial 2 μL injection, followed by 27 successive 10 μL injections, with a 210-s equilibration interval between each drop. To accurately isolate the heat of binding, a blank control experiment was performed by titrating the EPI solution into ultrapure water under identical conditions. The raw calorimetric data was baseline-corrected by subtracting the blank experimental data. The resulting binding isotherm was ultimately fitted to a Single-site binding model using the manufacturer's integrated Origin software to determine the specific thermodynamic parameters. The biophysical interactions and subsequent structural alterations between the RNAP enzyme and the EPI compound were evaluated using advanced spectroscopy. Purified RNAP at a standardized concentration was co-incubated with varying concentrations of EPI, alongside vancomycin as a negative control, for 30 min. The secondary multilinear structural conformation of the protein complexes was analyzed by measuring polarized light absorbance using a circular dichroism spectrometer at room temperature. Complementary UV-visible absorbance spectra of the mixtures were continuously recorded at predetermined time points (0 to 6 h) to track kinetic alterations in the characteristic absorption peaks of aromatic amino acids (approximately 280 nm) and the broader complex structure (approximately 590 nm).

### 4.5 Bacterial metabolomics assay

At the end of the co-culture of the drug and *E. coli* bacterial solution (volume of 200 μL, diluted to 10^5^ CFU mL^-1^) in 96-well plates, the supernatant of the bacterial solution was removed by centrifugation at 4000 g for 5 min, and then the precipitate was washed two times with pre-cooled PBS solution, and centrifuged for 5 min at 4000 g at 4°C. The enriched precipitates were systematically processed for downstream mass spectrometry-based metabolomic profiling to quantify differentially expressed genes, essential downstream cofactors and to conduct KEGG signaling pathway enrichment mapping focused on nucleic acid and energy metabolism. To identify significantly altered metabolic pathways, KEGG pathway enrichment analysis was performed. To strictly control the false discovery rate during multiple hypothesis testing, the raw p-values were adjusted using the Benjamini-Hochberg (BH) correction method. Only pathways with an FDR-adjusted *p*-value <0.05 were considered statistically significant.

To validate the transcriptomic perturbations associated with EPI treatment, quantitative real-time PCR was performed. *E. coli* suspensions (1x10^5^ CFU/mL) were co-incubated with EPI (8 μg/mL) or an equivalent volume of PBS (control) at 37°C. Following the incubation period, bacterial cells were harvested by centrifugation at 4000 *g* for 5 min at 4°C. Total bacterial RNA was extracted using the TRIzol Reagent (Invitrogen, USA) combined with enzymatic lysis (lysozyme, 1 mg/mL) to ensure complete disruption of the bacterial cell wall. The concentration and purity of the extracted RNA were quantified utilizing a NanoDrop 2000 spectrophotometer (Thermo Fisher Scientific, USA), ensuring an A260/A280 ratio between 1.8 and 2.0.

Subsequently, 1 μg of total RNA was reverse transcribed into complementary DNA (cDNA) using a PrimeScript™ RT reagent Kit with gDNA Eraser (Takara Bio, Japan) according to the manufacturer's protocols. The quantitative PCR reactions were assembled using the SYBR® Premix Ex Taq™ II kit (Takara Bio, Japan) and executed on a LightCycler 480 Real-Time PCR System (Roche, Switzerland). The thermal cycling conditions consisted of an initial denaturation at 95°C for 30 s, followed by 40 cycles of 95°C for 5 s and 60°C for 30 s. Melt curve analysis was routinely performed to verify the specificity of the amplified products. The expression levels of the target genes (*rpoB*, *atpA*, *ndh*, and *argF*) were normalized to the endogenous reference gene (*16S rRNA*). The specific primer sequences are detailed in the Supporting Information (Table S1).

### 4.6 In vitro cytocompatibility and hemolysis assays

The inherent biocompatibility of the screened compounds was initially validated using NIH3T3 murine fibroblasts and subsequently evaluated on pulmonary alveolar epithelial cells to accurately model local lung tissue tolerance. Cells were seeded in 96-well plates and co-cultured with escalating concentrations of the drug for 1 and 3 days. Cellular viability was quantitatively assessed using a Cell Counting Kit-8 (CCK-8) assay, measuring absorbance at 450 nm with strict background subtraction. Furthermore, to visually evaluate cytocompatibility, the pulmonary alveolar epithelial cells were subjected to Live/Dead fluorescence staining utilizing Calcein-AM and Propidium Iodide (PI). Following the designated co-incubation periods, the cells were stained according to the manufacturer's protocols and immediately visualized using a fluorescence inverted microscope to differentiate live cells (intact membranes, green fluorescence) from dead cells (compromised membranes, red fluorescence). To evaluate hemocompatibility for systemic administration, erythrocyte suspensions were exposed to EPI, alongside PBS (negative control) and Triton X-100 (positive control, 100% lysis). Samples were centrifuged, and macroscopic evaluation of the supernatant clarity and erythrocyte precipitation was utilized to confirm negligible hemolytic activity.

### 4.7 Isolation of UC-MSC exosomes and EPI encapsulation

Exosome Isolation: Human umbilical cord mesenchymal stem cells (UC-MSCs) were cultured in standard complete media until 70-80% confluence, after which the media was replaced with exosome-depleted fetal bovine serum (FBS) medium. After 48 h, the conditioned medium was collected and subjected to differential centrifugation to isolate exosomes. First, the medium was centrifuged at 300 × g for 10 min, followed by 2000 × g for 20 min at 4°C to remove dead cells and apoptotic bodies. The supernatant was then centrifuged at 10,000 × g for 30 min to eliminate larger microvesicles and cell debris. Finally, the clarified supernatant was subjected to ultracentrifugation at 100,000 × g for 70 min at 4°C (Optima XE-100, Beckman Coulter) to pellet the exosomes. The resulting exosome pellet was washed with sterile PBS, ultracentrifuged again under the same conditions, and resuspended in PBS for immediate use or stored at −80°C.

Preparation of Exo/EPI: EPI was loaded into the purified UC-MSC exosomes utilizing a combined sonication and incubation method. Briefly, 1 mg of purified exosomes (quantified via BCA protein assay) was mixed with 1 mg of Hydrochloride Epirubicin (EPI) in 1 mL of PBS. The mixture was subjected to probe sonication at 20% amplitude with a 3 s on/5 s off cycle for a total of 3 min in an ice bath to transiently permeabilize the exosome membranes. Following sonication, the mixture was incubated at 37°C for 1 h to allow for membrane recovery and maximal drug entrapment. To remove free, unencapsulated EPI, the suspension was transferred to a 100 kDa molecular weight cutoff (MWCO) ultrafiltration spin filter (Amicon Ultra, Millipore) and centrifuged at 4000 × g for 15 min, followed by two subsequent washes with PBS.

Encapsulation Efficiency: The concentration of the encapsulated EPI was determined by measuring the absorbance of the disrupted Exo/EPI at 480 nm using a UV-Vis spectrophotometer. The encapsulation efficiency (EE%) and loading capacity (LC%) were calculated using the following standard formulas: EE% = (Weight of encapsulated EPI/Initial weight of EPI added) × 100%; LC% = (Weight of encapsulated EPI/Total weight of Exo/EPI) × 100%.

Physicochemical Characterization and *In vitro* Drug Release: The surface charge (Zeta potential) of empty exosomes and Exo/EPI was measured using a Malvern Zetasizer Nano ZS at 25°C. To determine the *in vitro* drug release profile, a standard dialysis method was employed. Briefly, 2 mL of the Exo/EPI suspension was sealed in a dialysis bag (MWCO 14 kDa) and immersed in 30 mL of PBS (pH 7.4) with continuous shaking at 100 rpm at 37°C. At predetermined time intervals (1, 2, 4, 8, 12, 24, 48, and 72 h), 1 mL of the release medium was extracted and entirely replaced with an equal volume of fresh, pre-warmed PBS. The concentration of released EPI was quantified via UV-Vis spectrophotometry at 480 nm to generate the cumulative release curve.

### 4.8 In vivo murine models of pneumonia

All *in vivo* protocols utilized 4–6-week-old male C57BL/6J mice. Following a 3-day acclimatization period, severe acute pneumonia was induced via aerosolized inoculation. Briefly, mice were challenged with 50 μL of MDR *E. coli* suspension at a strictly controlled inoculum size of 1 × 10^7^ CFU per mouse. Once severe acute pneumonia was successfully established (24 h post-infection), therapeutic formulations were administered using an aerosolized inhalation chamber apparatus to ensure uniform distribution throughout the pulmonary microenvironment. Clinical progression was monitored through daily survival tracking.

To evaluate the baseline efficacy and systemic profile of the free drug, mice were randomly distributed into three specific cohorts: an uninfected healthy control, an untreated negative control (MDR *E. coli* only, treated with PBS), and a free EPI treatment group. The free EPI formulation was administered at an effective dosage of 5 mg/kg body weight. To correlate therapeutic efficacy with systemic clearance, tissue harvesting for ex vivo metabolic kinetics was strictly timed at 12 h and 48 h post-treatment. At 1, 2, and 4 days of post-treatment, animals were euthanized to harvest pulmonary tissues and major systemic organs (heart, liver, spleen, kidneys). Fresh lung homogenates were subjected to agar plate spreading for precise CFU quantification of bacterial clearance. Harvested tissues were fixed and sectioned for Hematoxylin and Eosin (H&E) staining, Masson's trichrome staining, Giemsa staining, and immunohistochemical evaluation of inflammatory factors. Additionally, routine hematological analysis was conducted to monitor potential systemic myelosuppression.

In a separate experiment designed to validate the dual 'kill-repair' functionality of the engineered delivery system against refractory infections, traditional standard-of-care antibiotic controls were purposefully excluded. Infected mice were randomly distributed into three distinct interventional cohorts: a PBS vehicle control (baseline damage), a free exosome (Exo) group (baseline immunomodulation), and the Exo/EPI nanoplatform treatment group. The Exo/EPI formulation was administered at an equivalent effective EPI dosage of 5 mg/kg body weight. At 1, 3, and 5 days of post-treatment, animals were euthanized. Pulmonary tissues were harvested, fixed, and sectioned specifically for H&E staining to evaluate inflammatory infiltration, Masson's trichrome staining for collagen deposition, and TUNEL assays for cellular apoptosis.

To quantitatively evaluate extracellular matrix remodeling and physiological collagen deposition, digital image analysis of the Masson's trichrome-stained histological sections was performed. Five randomly selected, non-overlapping fields of view were acquired for each biological replicate (n = 5) using brightfield microscopy. The images were exported to ImageJ for semi-quantitative analysis. The Color Deconvolution plugin (utilizing the "Masson Trichrome" vector) was employed to computationally isolate the blue-stained collagen channels from the red counterstain. A uniform global threshold was applied across all images to segment the isolated collagen signal, and the Collagen Volume Fraction (CVF) was calculated as the percentage of the positively stained blue area relative to the total viable tissue area in the field of view.

### 4.9 Statistical analysis

All quantitative experimental data were processed and analyzed using standard statistical software. Intergroup variance and statistical significance were determined by utilizing a one-way analysis of variance (ANOVA). Results across all assays are expressed as the mean value accompanied by the standard deviation (Mean ± SD).

## Data availability statement

The data that support this study are available on request from the corresponding author.

## Ethics approval and consent to participate

All animal experiments described in this study were conducted in accordance with protocols approved by the Center for Food and Drug Safety Evaluation and Experimental Animal Welfare and Ethics - IACUC (TOP-2SL-GM250712).

## CRediT authorship contribution statement

**Yiming Xiang:** Conceptualization, Data curation, Formal analysis, Funding acquisition, Investigation, Methodology, Validation, Writing – original draft, Writing – review & editing. **Ziya Gong:** Investigation, Methodology. **Juying Liu:** Investigation, Methodology. **Yizhou Zhu:** Resources, Validation. **Congyang Mao:** Formal analysis, Methodology, Visualization. **Can Ai:** Methodology, Visualization. **Chaofeng Wang:** Formal analysis, Investigation, Visualization. **Xiaofei Yang:** Resources, Visualization. **Xiangmei Liu:** Conceptualization, Formal analysis, Funding acquisition, Supervision, Writing – review & editing. **Kelvin W.K. Yeung:** Conceptualization, Supervision. **Shuilin Wu:** Conceptualization, Formal analysis, Funding acquisition, Supervision, Writing – review & editing.

## Declaration of competing interest

Kelvin W. K. Yeung is an associate editor for Bioactive Materials and was not involved in the editorial review or the decision to publish this article and Shuilin Wu is an editorial board member for Bioactive Materials and was not involved in the editorial review or the decision to publish this article. All authors declare that there are no competing interests.
