# Supplementary material for: Exosomes delivering a high-throughput-screened RNA polymerase inhibitor for highly effective therapy of multidrug-resistant bacterial infected pneumonia
Source: Bioact Mater. 2026 Jul 16;66:623–37. doi: 10.1016/j.bioactmat.2026.07.023 (PMC13400349; doi:10.1016/j.bioactmat.2026.07.023)
Supplement: Multimedia component 1 [file mmc1.docx]

***Supporting Information***

**Exosomes delivering a high-throughput-screened RNA polymerase inhibitor for highly effective therapy of multidrug-resistant bacterial infected pneumonia**

Yiming Xiang ^a,b^, Ziya Gong ^a^, Juying Liu ^a^, Yizhou Zhu ^b^, Congyang Mao ^b^, Can Ai ^b^, Chaofeng Wang ^c^, Xiaofei Yang ^a^, Xiangmei Liu ^c,*^, Kelvin W. K. Yeung ^b,**^, Shuilin Wu ^d,***^

*^a^ Hubei Key Laboratory of Medical Information Analysis and Tumor Diagnosis & Treatment, Key Laboratory of Cognitive Science, College of Biomedical Engineering, South-Central Minzu University, Wuhan, 430074, China*

*^b^ Department of Orthopaedics & Traumatology, Li Ka Shing Faculty of Medicine, The University of Hong Kong, Pokfulam, Hong Kong, 999077, China*

*^c^ School of Life Science and Health Engineering, Hebei University of Technology, Tianjin, 300401, China*

*^d^ School of Materials Science and Engineering, Peking University, Beijing, 100871, China*

* Corresponding Authors. School of Life Science and Health Engineering, Hebei University of Technology, Tianjin, 300401, China.

** Corresponding Authors. Department of Orthopaedics & Traumatology, Li Ka Shing Faculty of Medicine, The University of Hong Kong, Pokfulam, Hong Kong, 999077, China.

*** Corresponding Authors. School of Materials Science and Engineering, Peking University, Beijing, 100871, China.

E-mail: [liuxiangmei1978@163.com](mailto:liuxiangmei1978@163.com) (X. Liu) [wkkyeung@hku.hk](mailto:wkkyeung@hku.hk) (K. Yeung), [slwu@pku.edu.cn](mailto:slwu@pku.edu.cn) (S. Wu)


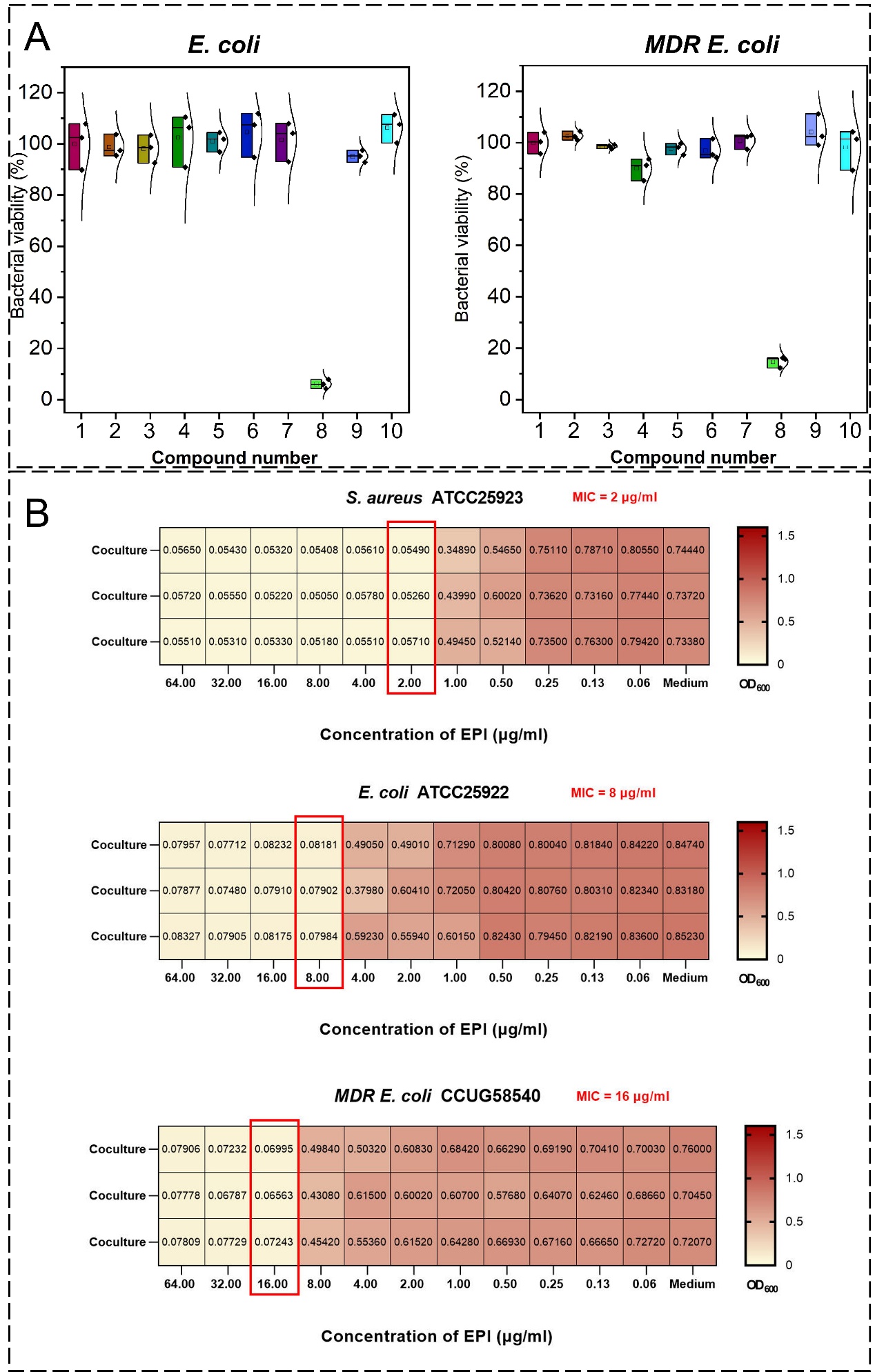


**Fig. S1.** Quantitative analysis of the antibacterial efficacy of candidate compounds. (A) Bacterial viability (%) of *E. coli* (left) and MDR *E. coli* (right) was determined by quantifying colony-forming units (CFUs) from the plate spread assays. Consistent with the results shown in Fig. 2D, EPI (Compound 8) exhibited the most potent antibacterial activity, achieving an inhibition rate exceeding 80% against both standard and MDR strains. In contrast, the other nine screened compounds demonstrated insufficient antibacterial performance against these Gram-negative pathogens. Data are presented with individual data points and distribution curves to illustrate experimental consistency. (B) Minimum Inhibitory Concentration (MIC) assays of EPI against targeted bacterial strains. Heatmaps displaying the optical density (OD600) of bacterial cultures following co-incubation with serially diluted EPI concentrations (ranging from 0.06 to 64.00 μg/mL). The red boxes indicate the determined MIC values, defined as the lowest drug concentration that effectively inhibits visible bacterial growth. The established MIC values for EPI are 2 μg/mL against *S. aureus*, 8 μg/mL against standard *E. coli*, and 16 μg/mL against multidrug-resistant MDR *E. coli*.


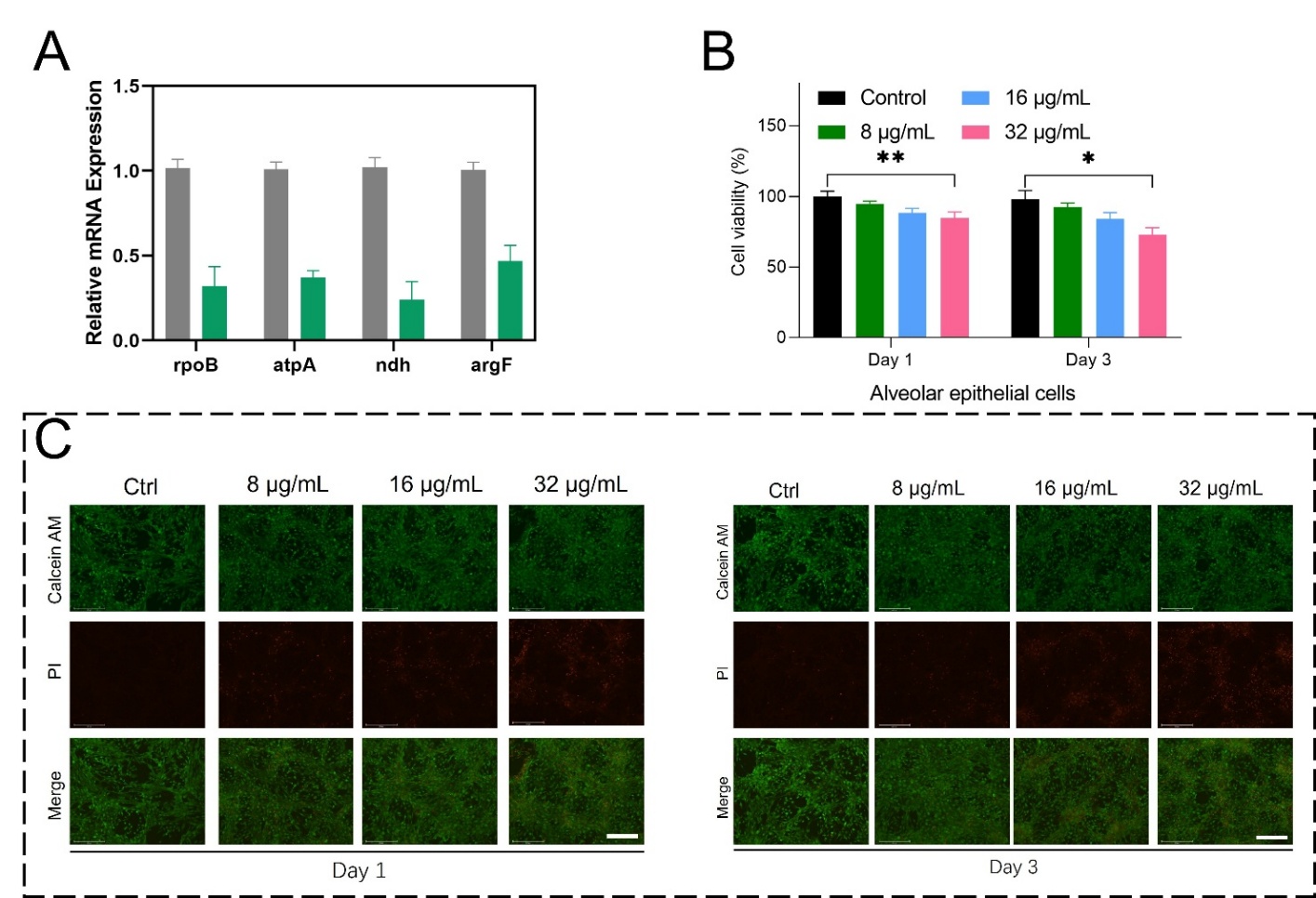


**Fig. S2.** (A) RT-qPCR validation of transcriptional and metabolic suppression in *E. coli* following EPI treatment. The relative mRNA expression levels of key genes associated with transcription (*rpoB*), oxidative phosphorylation/ATP synthesis (*atpA*), respiration (*ndh*), and arginine biosynthesis (*argF*) were quantified after 12 hours of co-incubation with EPI (8 μg/mL). Data are presented as mean ± SD (n = 5 independent biological replicates). (B) Representative Live/Dead fluorescence staining (Calcein-AM/PI) images of alveolar epithelial cells treated with escalating concentrations of EPI at Day 1 and Day 3. Green fluorescence indicates viable cells, while red fluorescence indicates dead cells (Scale bar = 650 μm). (C) Quantitative cell viability assessed via CCK-8 assay after 1 and 3 days of co-incubation, demonstrating sustained high viability at the therapeutic micro-dose of 8 μg/mL. Statistical significance was determined utilizing a one-way ANOVA (*p < 0.05, **p < 0.01 compared to the control group)

**
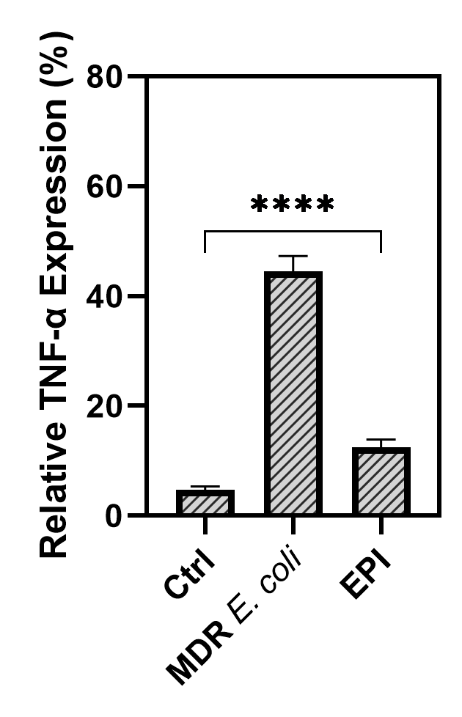

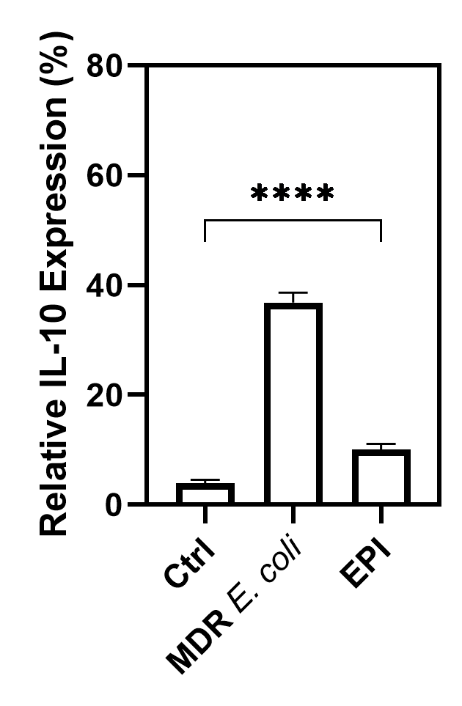
**

**Fig. S3.** Quantitative expression data of immunohistochemical. Statistical significance was determined utilizing a one-way ANOVA followed by Tukey's post hoc test (p < 0.0001 compared to the untreated MDR *E. coli* control group).

**
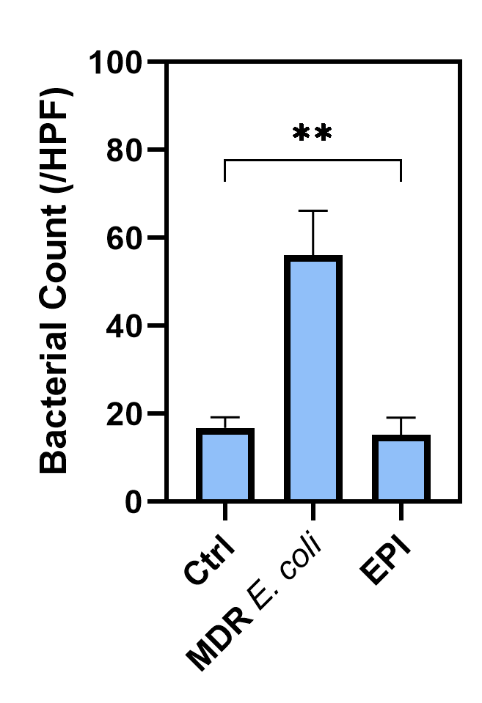
**

**Fig. S4.** Quantitative expression data of Giemsa staining. Statistical significance was determined utilizing a one-way ANOVA followed by Tukey's post hoc test (p < 0.001, compared to the untreated MDR *E. coli* control group).


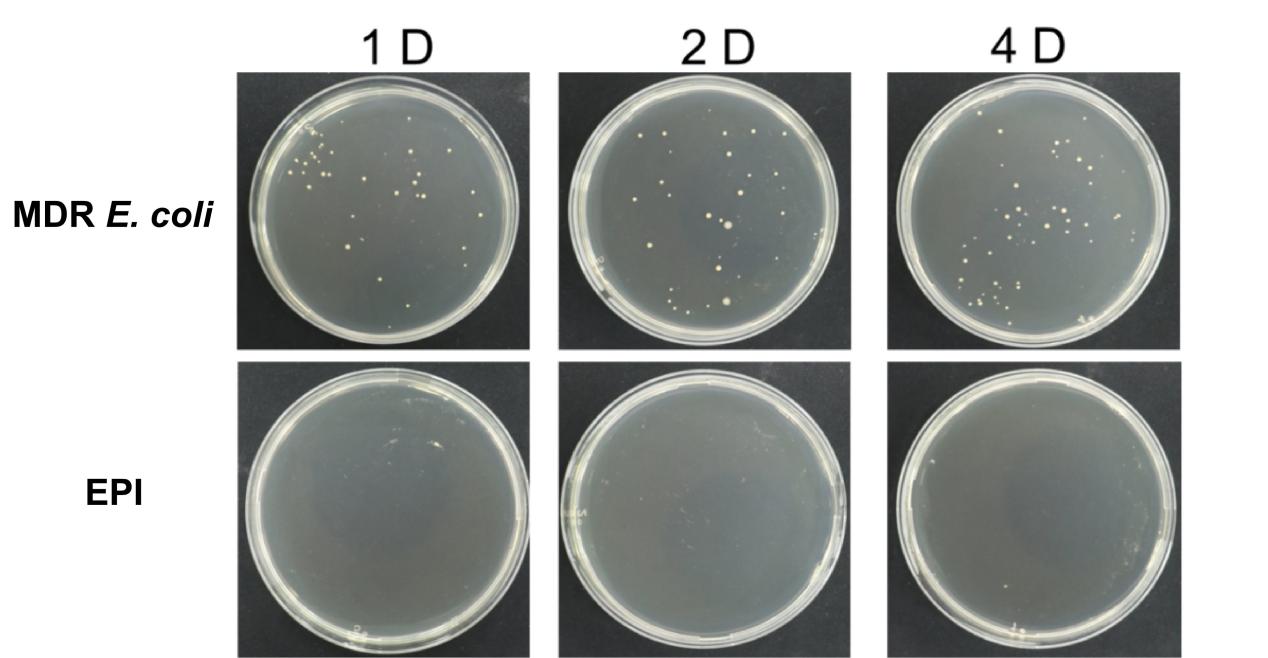


**Fig. S5.** Visual validation of pulmonary bacterial clearance via tissue homogenization assays. Representative images of spread plates generated from lung tissue homogenates at 1, 2, and 4 days post-infection. While significant bacterial colonies persisted in the MDR *E. coli*-infected negative control group across all time points, the EPI-treated group showed complete clearance of bacterial pathogens from the lung tissue.


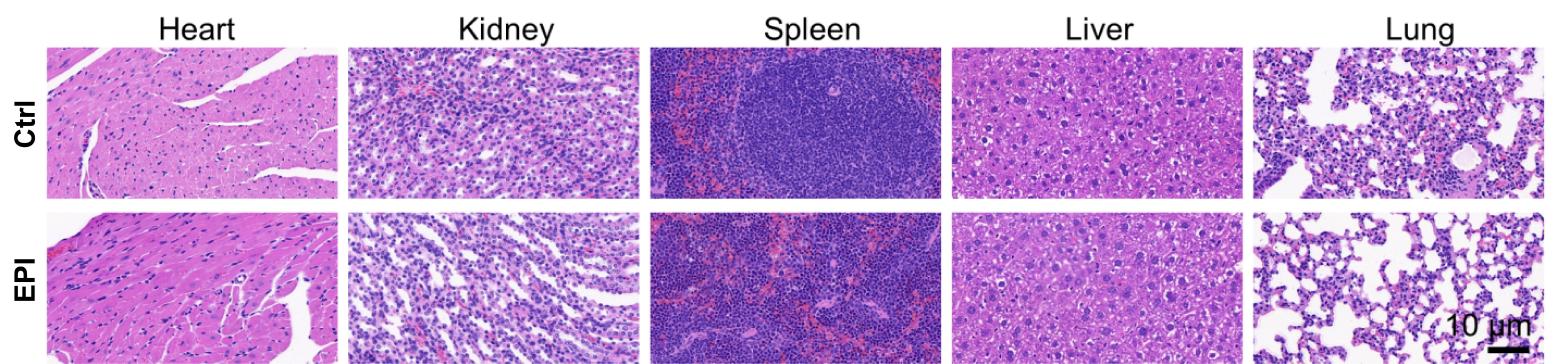


**Fig. S6.** Histopathological biosafety evaluation of major systemic organs. H&E-stained sections of the heart, kidney, spleen, liver, and lung from mice treated with PBS (Control) and EPI. No significant tissue abnormalities, inflammatory infiltration, or morphological lesions were observed in the major organs of the EPI group, suggesting a high degree of systemic biosafety and negligible off-target toxicity.


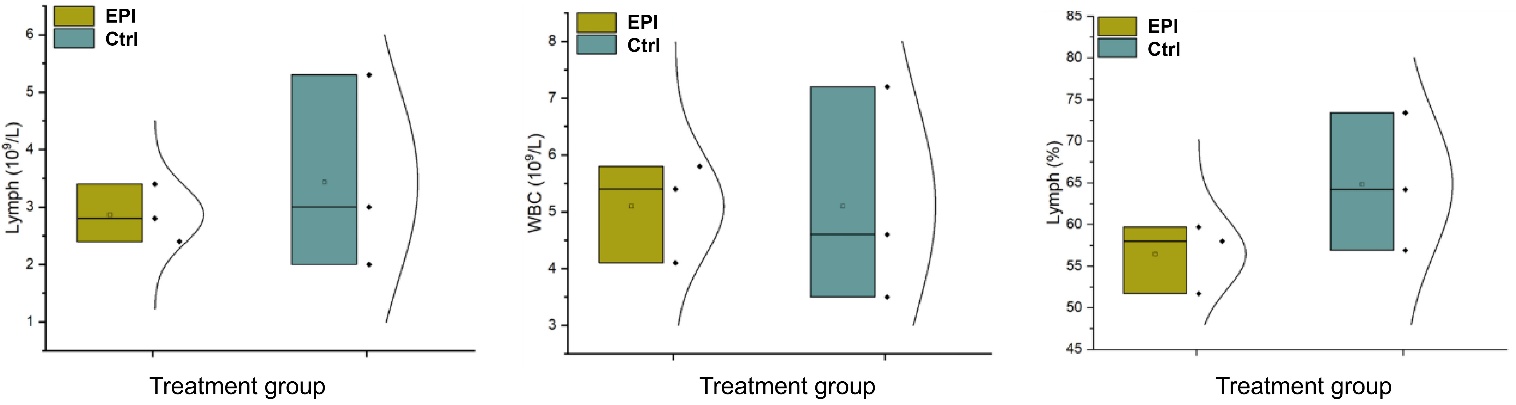


**Fig. S7.** Hematological parameters illustrating EPI-induced myelosuppression. Statistical analysis of routine blood data, including white blood cell (WBC) counts, lymphocyte (Lymph) counts, and lymphocyte percentages, recorded after 2 days of therapeutic intervention with free EPI. The drastic decline in WBCs and lymphocytes highlights the severe, systemic myelosuppressive toxicity associated with free EPI administration, reinforcing the clinical need for localized exosome-mediated delivery strategies to avoid these adverse effects.

**

**

**Fig. S8.** Encapsulation parameters of the Exo/EPI nanoplatform. Quantitative analysis of Encapsulation Efficiency (EE%) and Loading Capacity (LC%) for EPI loaded into UC-MSC exosomes. Data are presented as mean ± SD (n = 3).

**

**

**Fig. S9.** Surface charge characterization. Zeta potential analysis of empty UC-MSC exosomes and Exo/EPI. The mild shift confirms drug incorporation while maintaining sufficient negative charge for colloidal stability. Data are presented as mean ± SD (n = 3).

**

**

**Fig. S10.** In vitro drug release kinetics. Cumulative release profile of EPI from the Exo/EPI nanocarrier evaluated over 72 hours via dialysis in PBS (pH 7.4) at 37 °C. The sustained-release pattern minimizes premature leakage, ensuring prolonged localized therapy. Data are presented as mean ± SD (n = 3).


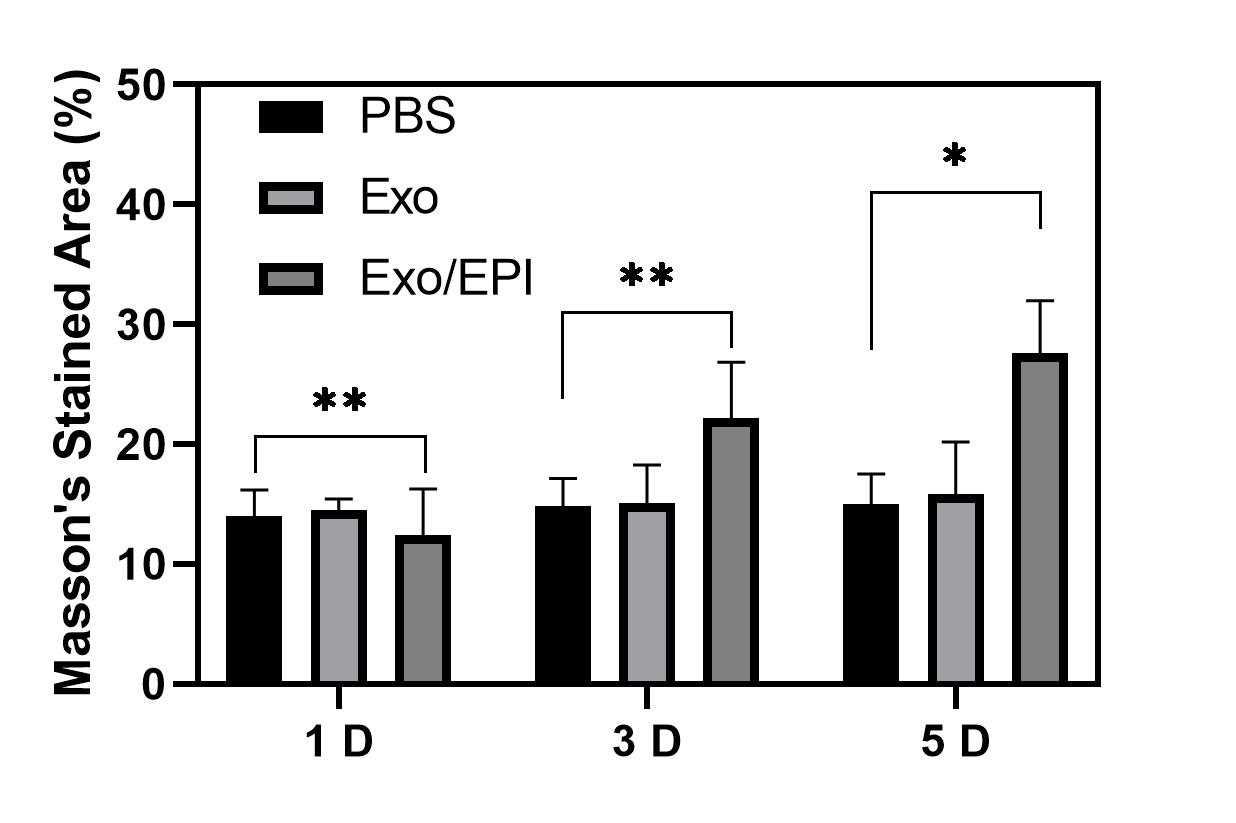


**Fig. S11.** Quantitative evaluation of the relative Masson's Stained Area (%). The digital image analysis demonstrates that treatment with the Exo/EPI nanoplatform significantly promotes collagen deposition by Day 3 and Day 5 compared to the untreated PBS control. This enhanced collagen volume fraction indicates active, physiological tissue regeneration and extracellular matrix repair mediated by the exosome delivery system, contrasting with the persistent tissue destruction observed in the infected PBS group. Statistical significance was determined using a two-way ANOVA (*p < 0.05, **p < 0.01).

**
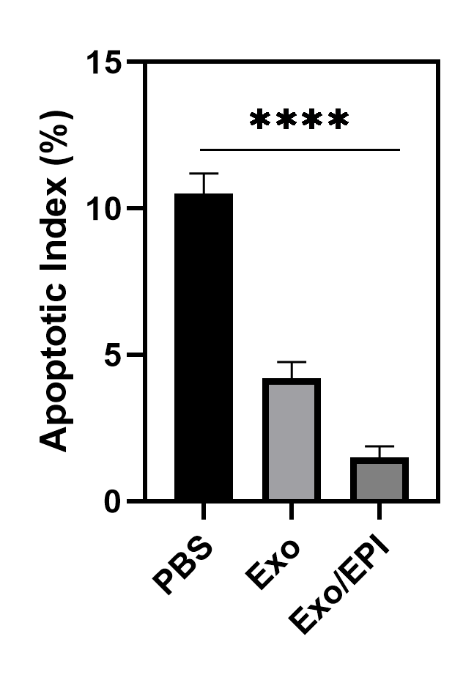
**

**Fig. S12.** Quantitative evaluation of Exo/EPI anti-apoptotic efficacy in vivo. Statistical analysis of the Apoptotic Index derived from TUNEL fluorescence staining of lung tissue sections following acute bacterial infection. The index represents the ratio of TUNEL-positive cells to total DAPI-stained nuclei. The Exo/EPI nanoplatform significantly reduced infection-induced cellular apoptosis compared to the PBS and free Exo treatment groups. Data are expressed as the mean ± SD (n = 5 independent fields of view per group). Statistical significance was determined utilizing a one-way ANOVA with Tukey's post hoc test (*p* < 0.0001).

**Table S1.** Primers utilized for quantitative real-time PCR (RT-qPCR) analysis.

| Gene Target | Forward Primer Sequence (5' → 3') | Reverse Primer Sequence (5' → 3') | Pathway / Function |
| --- | --- | --- | --- |
| 16S rRNA | CGGTCCAGACTCCTACGGG | TTACGCCCAGTAATTCCGATT | Internal reference control |
| rpoB | GCTCGCTTCGCCCACGTCA | CAGGCCAGCATCTTTGCCGC | RNA transcription machinery |
| atpA | GCACGAGATTCGTCAGGTTC | GCCACACCTGCACGGTAA | Oxidative phosphorylation (ATP synthesis) |
| ndh | TCGCTACTCGCTGGAATACG | CCGCTGGTACAGTTCGTGAT | NADH metabolism / Respiration |
| argF | CGTTAATGCGCCATGTTAC | CGCCCATCAGTTTACCTAC | Arginine biosynthesis |
